# Bacterial cytosolic proteins with a high capacity for Cu(I) that protect against copper toxicity

**DOI:** 10.1038/srep39065

**Published:** 2016-12-19

**Authors:** Nicolas Vita, Gianpiero Landolfi, Arnaud Baslé, Semeli Platsaki, Jaeick Lee, Kevin J. Waldron, Christopher Dennison

**Affiliations:** 1 Institute for Cell and Molecular Biosciences, Medical School, Newcastle University, Newcastle upon Tyne NE2 4HH, UK

## Abstract

Bacteria are thought to avoid using the essential metal ion copper in their cytosol due to its toxicity. Herein we characterize Csp3, the cytosolic member of a new family of bacterial copper storage proteins from *Methylosinus trichosporium* OB3b and *Bacillus subtilis*. These tetrameric proteins possess a large number of Cys residues that point into the cores of their four-helix bundle monomers. The Csp3 tetramers can bind a maximum of approximately 80 Cu(I) ions, mainly via thiolate groups, with average affinities in the (1–2) × 10^17^ M^−1^ range. Cu(I) removal from these Csp3s by higher affinity potential physiological partners and small-molecule ligands is very slow, which is unexpected for a metal-storage protein. *In vivo* data demonstrate that Csp3s prevent toxicity caused by the presence of excess copper. Furthermore, bacteria expressing Csp3 accumulate copper and are able to safely maintain large quantities of this metal ion in their cytosol. This suggests a requirement for storing copper in this compartment of Csp3-producing bacteria.

Cells do not typically store transition metal ions. The most notable exceptions are iron that is stored by ferritins^1^ and zinc and copper sequestration by metallothioneins (MTs)^2^. Copper is essential for almost all organisms yet can also be harmful due to its redox activity and the ability to bind to sites for other metals, particularly iron-sulfur clusters^3^. This has resulted in the evolution of intricate homeostatic systems that facilitate copper’s use as the cofactor for many important enzymes in both eukaryotes and prokaryotes^4^,^5^,^6^. The response to excess copper regularly involves a copper-exporting P-type ATPase^4^,^5^,^6^,^7^. Also present are the cytosolic copper metallochaperone ATOX1 (HAH1) in humans, Atx1 in yeast and CopZ in many bacteria, which bind Cu(I) tightly^4^,^5^,^6^,^8^,^9^,^10^,^11^, and are thought to deliver the metal to ATPases^4^,^5^,^6^. In addition to efflux systems, eukaryotes possess the cytosolic Cys-rich MTs^2^,^4^,^5^, which can fold around multiple Cu(I) ions forming thiolate-coordinated clusters^2^,^12^,^13^. Related copper-binding bacterial MTs have only been found to date in certain pathogenic mycobacteria^14^, and most bacteria are currently not thought to maintain copper in the cytosol^5^,^15^. The interplay between copper homeostasis in bacteria and humans is of medical importance as copper appears to be exploited as a weapon by the mammalian immune system to fight bacterial pathogens, and copper homeostasis genes provide a fitness advantage during infection^16^.

Methane oxidizing bacteria (methanotrophs) require large amounts of copper for the active site of the membrane-bound (particulate) methane monooxygenase (pMMO)^17^. Some methanotrophs, including *Methylosinus trichosporium* OB3b, can use a soluble iron sMMO when copper levels are low, with switchover regulated by copper^18^. MMOs and methanotrophs have great potential for biotransformations giving a range of products, in bioremediation and for mitigating the release of methane, a potent greenhouse gas, to the atmosphere^19^,^20^,^21^. Understanding copper handling and the expression of either pMMO or sMMO is essential for all these biotechnological applications. Recently, a new family of bacterial copper storage proteins, the Csps, has been discovered in *M. trichosporium* OB3b^22^. Three Csps are present in this model methanotroph; two (*Mt*Csp1 and *Mt*Csp2) with predicted twin arginine translocase (Tat) targeting signal peptides are exported from the cytosol. *Mt*Csp1 has been characterized and is a tetramer that can bind up to 52 Cu(I) ions mainly via Cys residues^22^. Deletion of the genes for *Mt*Csp1 and *Mt*Csp2 increases the rate of switchover from pMMO to sMMO in *M. trichosporium* OB3b, implicating these proteins in the storage of copper for methane oxidation^22^. *M. trichosporium* OB3b also possesses a Csp3 that lacks a signal peptide and must be cytosolic, with homologues of this protein present in a wide range of bacteria (Supplementary Fig. S1).

pMMO is housed on specialized intracytoplasmic membranes^23^ that are either contiguous with the periplasm or form discrete compartments and thus pMMO may be a rare example of a bacterial cytoplasmic copper enzyme. *M. trichosporium* OB3b possesses homologues of the copper-efflux ATPase CopA^7^ and the copper-dependent transcriptional activator CueR^24^, but lacks CopZ^25^. However, this methanotroph produces methanobactin (mbtin), a small modified peptide that is secreted and can sequester copper^18^,^26^,^27^,^28^,^29^. Copper-loaded mbtin is imported by MbnT, a TonB-dependent transporter^30^, but other copper uptake mechanisms are also present^30^,^31^. It has been suggested that CopD is an inner membrane copper importer in *M. trichosporium* OB3b, and may play a role in non-mbtin-bound copper uptake^32^. Although both mbtin and CopD are implicated in delivering copper to the cytosol in *M. trichosporium* OB3b, how this is safely handled is currently unknown.

A cytosolic Csp3 is also present in *Bacillus subtilis*, whose metal ion homeostasis has been studied in detail^33^,^34^,^35^. This model Gram positive bacterium possesses the copper-dependent transcriptional activator CsoR^36^,^37^,^38^, which regulates expression of both CopA and CopZ (*Bs*CopZ)^36^. The deletion of *copZ*, and particularly *copA*, increases sensitivity to elevated copper levels^39^,^40^, with the Δ*copA* strain exhibiting a 3-fold decrease in cellular copper content, whilst the absence of CopZ causes copper levels to increase 2.6 fold^40^. *B. subtilis* is known to possess two classes of copper-requiring enzymes; cytochrome oxidases^41^,^42^ and a multi-copper oxidase (CotA)^43^, located on the cytoplasmic membrane and spore surface respectively. A bioinformatics study also suggested the presence of the Cu, Zn superoxide dismutase SodC^44^. However, *in vitro* analysis of this protein, which lacks two of the copper ligands, demonstrates that it cannot bind copper and has no superoxide dismutase activity^45^. Regardless of the lack of a currently identified intracellular requirement for copper, a protein (YcnJ) with homology to both CopD and CopC, the latter a periplasmic copper metallochaperone found in Gram negative bacteria, at its C- and N-termini respectively has been suggested to be involved in copper uptake into the cytosol of *B. subtilis*^46^.

To understand the role of Csp3s in cytosolic copper-handling by Gram negative and Gram positive bacteria we have characterized the proteins from *M. trichosporium* OB3b (*Mt*Csp3) and *B. subtilis (Bs*Csp3). This has included investigating their Cu(I)-binding and release properties *in vitro*. Furthermore, the ability of *Bs*Csp3 to buffer copper and prevent toxicity *in vivo* has been analysed. Overall, these studies highlight significant functional diversity of the cytosolic Csp3s relative to their exported counterparts, and demonstrate that as in eukaryotes, Csp3-containing bacteria possess a storage mechanism in addition to efflux, as a response to copper toxicity. Csp3 provides a safe cytosolic source of copper, a finding of fundamental importance for understanding how bacteria utilize and respond to this metal.

## Results

### Protein quantification

Precise protein quantification is essential for the *in vitro* studies described herein with the Csp3s. The large number of Cys residues (18 in *Mt*Csp3 and 19 in *Bs*Csp3) allows the use of the 5,5′-dithiobis(2-nitrobenzoic acid) (DTNB, Ellman’s reagent) assay for precise quantification, but only for the unfolded proteins as there is little reactivity in the absence of denaturant (see Supplementary Methods). Unfolding is readily achieved for apo-*Mt*Csp3 using urea (Supplementary Fig. S2) and for apo-*Bs*Csp3 with guanidine hydrochloride (Supplementary Fig. S3), but not for either Cu(I)-protein (Supplementary Figs S2 and S3). Therefore, for Cu(I)-Csp3 samples the number of Cu(I) equivalents quoted is typically based on apo-protein concentrations determined by DTNB. Quantification of the Csp3s with the Bradford assay gives erroneous protein concentrations. Nevertheless, the values obtained with this assay are reproducible and the resulting Bradford:DTNB concentration ratio provides a method of detecting disulfide-bond formation in such Cys-rich proteins. A Bradford:DTNB ratio of 1.44 ± 0.13 (n = 77) was obtained for apo-*Mt*Csp3 not treated with dithiothreitol (DTT), with a ratio of 1.40 ± 0.12 (n = 39) for protein that was incubated overnight with DTT. For apo-*Bs*Csp3 the corresponding samples gave Bradford:DTNB ratios of 1.31 ± 0.11 (n = 49) and 1.25 ± 0.12 (n = 12) respectively. Treatment with DTT has no significant effect on thiol quantification demonstrating that the Cys residues of both apo-Csp3s are not readily susceptible to oxidation. This is confirmed by prolonged incubation in air after treatment with DTT (see Supplementary Methods) having almost no effect on the Bradford:DTNB ratio.

### The crystal structures of apo-Csp3s

**Figure 1:**
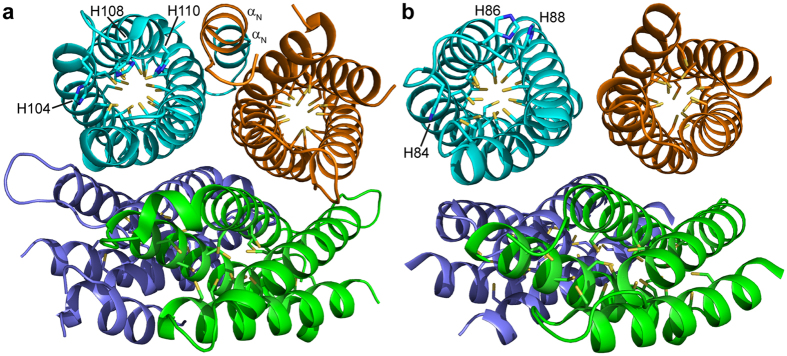
The structures of apo-Csp3s. The crystal structures of the (**a**) apo-*Mt*Csp3 (PDB file 5ARM) and (**b**) apo-*Bs*Csp3 (PDB file 5FIG) tetramers of four-helix bundles (rmsd of 1.09 Å for Cα atoms when comparing monomers, see Supplementary Fig. S4). The side chains of the Cys residues, which all point into the core of the bundles, are shown as sticks, as are the three His residues found at the opening of the bundle that is facing out in the cyan monomers. The additional N-terminal α-helix (α_N_) of apo-*Mt*Csp3 is labeled in two monomers (**a**). Pairs of anti-parallel four-helix bundles are rotated by ~35° in the tetramers, with contact areas between monomers ranging from ~1060 to 1410 Å^2^ and ~520 to 770 Å^2^ in apo-*Mt*Csp3 and apo-*Bs*Csp3 respectively.

**Figure 2:**
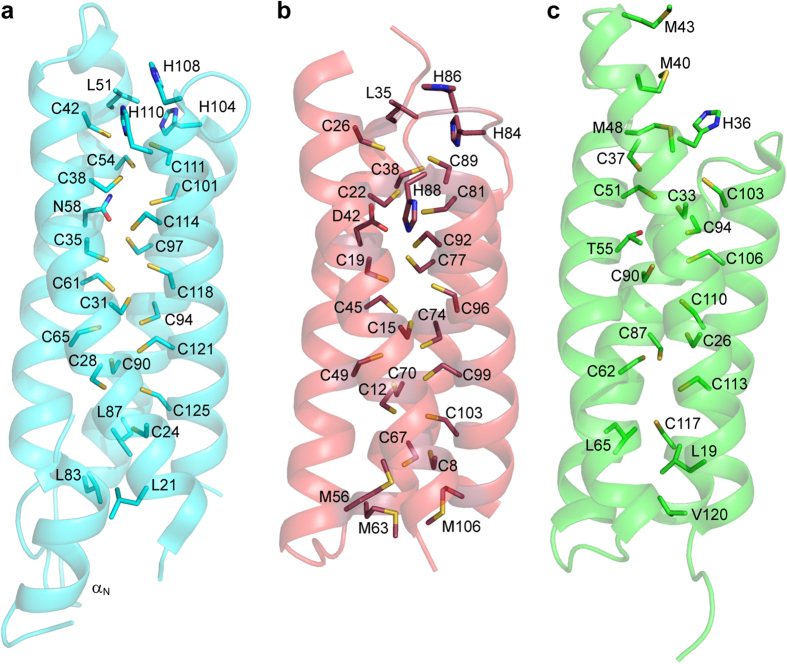
Comparison of apo-Csp monomers. The structures of the apo-*Mt*Csp3 (**a**), apo-*Bs*Csp3 (**b**) and apo*-Mt*Csp1^22^ (**c**) monomers. The side chains of the Cys residues, and the His residues at the more solvent-exposed end of the Csp3 four-helix bundles (*Bs*Csp3 also has His33 and His34 in this region from the loop between α1 and α2 that are not shown), are represented as sticks, as are His36 and the Met residues at the opening of *Mt*Csp1. The side chains of Leu51 (*Mt*Csp3, **a**) and Leu35 (*Bs*Csp3, **b**) that correspond to Met48 in *Mt*Csp1 (**c**) and Asn58 of *Mt*Csp3 (**a**) and the analogous Asp42 and Thr55 of *Bs*Csp3 (**b**) and *Mt*Csp1 (**c**) respectively, are also shown. Contributing to the hydrophobic end of the *Mt*Csp3 bundle (**a**) are side chains from three Leu residues (Leu21, Leu83 and Leu87). In *Bs*Csp3 (**b**) Cys67 is found in place of Leu87 and hydrophobicity in this region is primarily provided by Met56, Met63 and Met106, and by residues such as Leu19, Leu65 and Val120 in *Mt*Csp1 (**c**).

In the crystal structure, apo-*Mt*Csp3 is a four-helix bundle consisting of ~73% α-helical secondary structure (Figs 1a and 2a), very similar to that calculated (76.0 ± 2.8%, n = 10) from far-UV circular dichroism (CD) spectra (Supplementary Fig. S5). Apo-*Mt*Csp3 is a tetramer in the crystal (Fig. 1a), and in solution at higher protein concentrations (above ~80 μM) elutes from a gel-filtration column largely as a single peak with an apparent molecular weight (n = 31) of 45.2 ± 4.1 kDa (Supplementary Fig. S5). The discrepancy between this and the actual molecular weight of the *Mt*Csp3 tetramer (58.1 kDa) is due to the N-terminal α-helix (α_N_), which does not significantly increase the volume of the tetramer (Fig. 1a). The 18 Cys residues, all originating from α-helices, point into the core of the four-helix bundle of an apo-*Mt*Csp3 monomer and none are involved in disulfide bonds (Figs 1a and 2a), consistent with the Bradford:DTNB ratio data above. The crystal structure of apo-*Bs*Csp3 has also been determined (Figs 1b and 2b) and the protein is a four-helix bundle like that of apo-*Mt*Csp3 (the α-helical content in the structure is ~81%, very similar to the value of 83.1 ± 2.5% (n = 3) obtained from far-UV CD measurements, Supplementary Fig. S5). Apo-*Bs*Csp3 also forms a tetramer, but α_N_ is missing (Fig. 1b and Supplementary Fig. S4) and consequently the apparent molecular weight in solution (46.0 ± 1.8 kDa, n = 8, Supplementary Fig. S5) matches that expected (47.4 kDa). The 19 Cys residues, also all from α-helices, point into the core of the bundle of apo-*Bs*Csp3, with evidence of partial disulfide bond formation only between Cys38 and Cys81 in the crystal (Fig. 2b and Supplementary Fig. S4). In both Csp3 structures three highly conserved His residues (Supplementary Fig. S1) are found at the more solvent exposed end of the four helix bundle (Figs 1 and 2a,b).

### Cu(I)-binding stoichiometry of Csp3s

**Figure 3:**
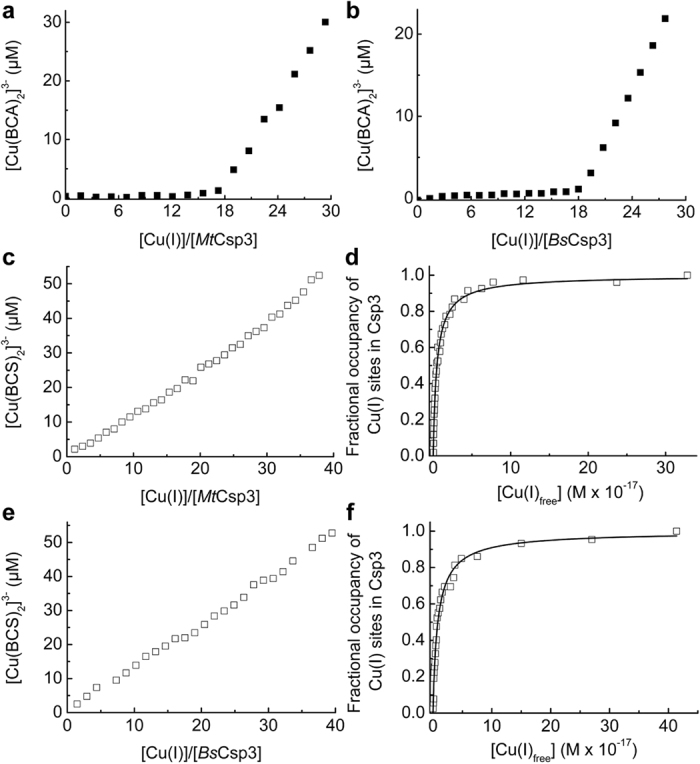
Cu(I)-binding stoichiometries and estimated average Cu(I) affinities of the Csp3s. Plots of [Cu(BCA)_2_]^3−^ concentration against the [Cu(I)]/[Csp3] ratio (for a monomer) of (**a**) mixtures of apo-*Mt*Csp3 (2.53 μM) and Cu(I) in the presence of 100 μM BCA (21 h incubation) and (**b**) for the titration of Cu(I) into apo-*Bs*Csp3 (2.34 μM) in the presence of 93.7 μM BCA. (**c**) A plot of [Cu(BCS)_2_]^3−^ concentration against the [Cu(I)]/[*Mt*Csp3] ratio (for a monomer) for mixtures of apo-protein (2.50 μM) and Cu(I) in the presence of 121 μM BCS (43 h incubation). (**d**) Fractional occupancy of Cu(I)-binding sites in *Mt*Csp3 (the maximum calculated occupancy is 16.9 Cu(I) equivalents per monomer in this experiment) at different concentrations of free Cu(I) from the data in (**c**). (**e**) A plot of [Cu(BCS)_2_]^3−^ concentration against the [Cu(I)]/[*Bs*Csp3] ratio (for a monomer) for mixtures of apo-protein (2.50 μM) and Cu(I) in the presence of 120 μM BCS (95 h incubation). (**f**) Fractional occupancy of Cu(I)-binding sites in *Bs*Csp3 (the maximum calculated occupancy is 18.4 Cu(I) equivalents per monomer) at different concentrations of free Cu(I) from the data in (**e**). All experiments were performed in 20 mM 4-(2-hydroxyethyl)piperazine-1-ethanesulfonic acid (Hepes) plus 200 mM NaCl at pH 7.5 and the solid lines in (**d**) and (**f**) show fits of the data to the nonlinear Hill equation giving average dissociation constants for Cu(I), *K*_Cu_, of (5.0 ± 0.1) × 10^−18^ M and (8.2 ± 0.3) × 10^−18^ M and Hill coefficients of 0.94 ± 0.02 and 0.92 ± 0.04 respectively.

Cu(I) titrations, monitored using the formation of (S)Cys → Cu(I) ligand-to-metal charge transfer bands below 350 nm^12^,^22^,^47^, give approximate Cu(I)-binding stoichiometries for monomers of 15 to 20 (n = 13) and 17 to 21 (n = 8) for *Mt*Csp3 (Supplementary Fig. S6) and *Bs*Csp3 (Supplementary Fig. S7) respectively. More precise Cu(I)-binding stoichiometries are obtained in the presence of a relatively small excess (~40-fold) of the high-affinity chromophoric Cu(I) ligand bicinchoninic acid (BCA, log*β*_2_ = 17.7 (ref. 48)), showing that under these conditions *Mt*Csp3 (n = 8) and *Bs*Csp3 (n = 10) bind all Cu(I) until 17.9 ± 1.0 (Fig. 3a) and 19.6 ± 0.8 (Fig. 3b) equivalents respectively have been added per monomer. Cu(I)-thiolate clusters, such as those found in MTs, typically give rise to luminescence in the 500 to 700 nm region^12^,^14^ due to solvent protected Cu(I)-Cu(I) interactions^12^,^49^,^50^,^51^. Such luminescence is observed and increases until ~9 to 11 (Supplementary Fig. S6) and ~8 to 12 (Supplementary Fig. S7) equivalents of Cu(I) per monomer are added to *Mt*Csp3 (n = 8) and *Bs*Csp3 (n = 3) respectively. Emission then decreases to almost zero upon addition of further Cu(I), either due to structural modification of the original sites or an increase in their solvent exposure^49^,^50^,^51^.

### Crystal structure of Cu(I)-*Mt*Csp3

**Figure 4:**
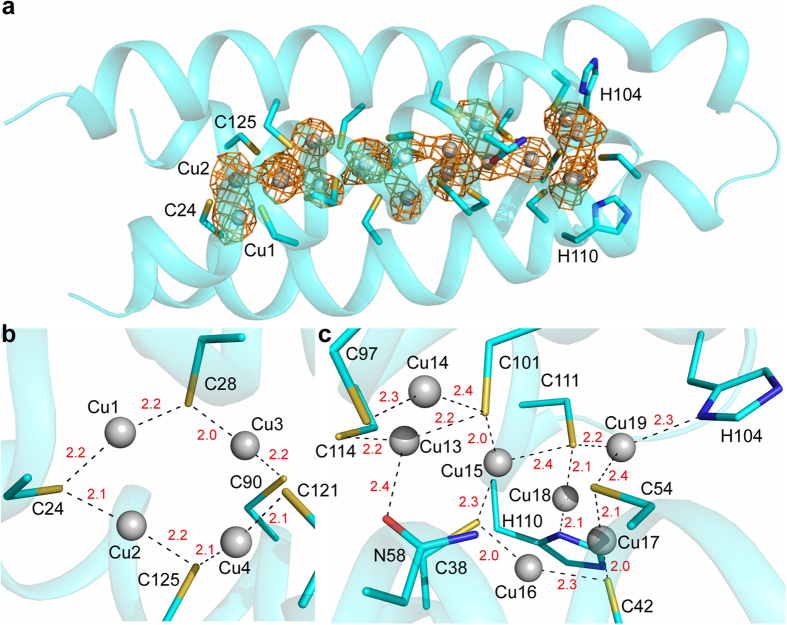
The structure of Cu(I)-*Mt*Csp3. (**a**) The crystal structure of an *Mt*Csp3 monomer (α_N_ omitted) binding 19 Cu(I) ions, including the anomalous difference density for copper contoured at 2σ (orange mesh). The Cu(I) ions (Cu1 to Cu19 correspond to Cu1130 to Cu1148 in PDB file 5ARN) are shown as light grey spheres and the side chains of Cys and other key residues as sticks. Detailed coordination of Cu(I) sites at the hydrophobic (**b**) and solvent exposed (**c**) ends of the bundle are shown with bond distances (Å) in red. At 15 of the 19 sites (Cu1 to Cu12, Cu14, Cu16 and Cu17) Cu(I) is coordinated solely by the thiolates of two Cys residues with Cu(I)–S(Cys) bond lengths and S(Cys)–Cu–S(Cys) angles ranging from 1.9 to 2.4 Å and 133° to 177°, respectively. The majority of the Cys ligands bridge two Cu(I) ions, except for Cys101 and Cys111 whose thiolates bridge three Cu(I) ions (**c**). Cu15 is coordinated by three Cys residues with Cu(I)–S(Cys) bond lengths and S(Cys)–Cu–S(Cys) angles of 2.0 to 2.4 Å and 97° to 153°, respectively (**c**). At eight Cu(I) sites, such as Cu1, Cu4, Cu14 and Cu16, the two thiolates are provided by a CXXXC motif from a single α-helix. The remaining Cu(I) sites are coordinated by Cys residues from adjacent α-helices and largely alternate with those bound by CXXXC motifs throughout the core.

The structure of *Mt*Csp3 is hardly affected (rmsd 0.32 Å for 116 aligned Cα atoms) by fully loading the protein with Cu(I) (Fig. 4a and Supplementary Fig. S8), consistent with far-UV CD data (α-helical content of 74.8 ± 2.2%, n = 4, Supplementary Fig. S5). The same tetrameric arrangement is present for Cu(I)-*Mt*Csp3 as for the apo-protein, in agreement with gel-filtration studies that give an apparent molecular weight (n = 12) of 44.6 ± 1.8 kDa (Supplementary Fig. S5). The anomalous difference density for data collected just below the copper-edge identifies 19 copper ions within the core of the four-helix bundle of Cu(I)-*Mt*Csp3 bound predominantly by Cys residues (Fig. 4a), consistent with Cu(I)-binding data (Fig. 3a and Supplementary Fig. S6). *Bs*Csp3 has a similar, if not slightly greater, Cu(I) capacity (see above), probably due to the presence of one more Cys residue. Like *Mt*Csp3, Cu(I) binding to *Bs*Csp3 does not affect either its secondary (76.0 ± 2.6% α-helical content, n = 3, Supplementary Fig. S5) or quaternary (43.3 ± 0.8 kDa, n = 8, Supplementary Fig. S5) structure.

The Cu(I) ions are located along the core of the four-helix bundle of *Mt*Csp3 (Fig. 4a). The majority of the sites involve Cu(I) coordinated by the thiolates of two Cys residues in an arrangement (Fig. 4b,c) reminiscent of that found in Cu(I) metallochaperones^15^,^47^,^52^, Cu(I) sensors^24^,^37^, and also *Mt*Csp1^22^. In Cu(I)-MT most sites are coordinated by three Cys ligands^13^, but only Cu15 (Fig. 4c) is ligated by three thiolates in Cu(I)-*Mt*Csp3. There are sites with unusual Cu(I) coordination such as Cu13 (Fig. 4c), which not only involves ligation by Cys101 and Cys114 (Cu(I)–S(Cys) bond lengths of 2.2 Å and a S(Cys)–Cu–S(Cys) angle of 148°) but also the O^δ1^ of Asn58 at 2.4 Å (S(Cys)–Cu–O(Asn) angles of 103° to 109°). Furthermore, Cu18 and Cu19, located at the end of the four-helix bundle at which Cu(I) sites are more solvent exposed, both involve coordination by one of the His residues found in this region (Fig. 4c). Cu18 is bound by His110 (N^δ1^) and Cys111 with Cu(I)–ligand distances of 2.1 Å and a S(Cys)–Cu–N(His) angle of 165°, whilst there are two Cys ligands (Cys54 and Cys111) as well as the coordinating His104 (N^δ1^) at Cu19 (Cu(I)–ligand distances of 2.2 to 2.4 Å and bond angles of 105° to 135°). Numerous Cu(I)-Cu(I) interactions are present in the core of Cu(I)-*Mt*Csp3, with 18 of the sites being 2.5 to 2.8 Å from an adjacent Cu(I) ion, and Cu5, Cu7, Cu9 and Cu13 are involved in three Cu(I)-Cu(I) interactions of <2.8 Å.

### Average Cu(I) affinities of the Csp3s

To estimate average Cu(I) affinities of the Csp3s the chromophoric ligand bathocuproine disulfonate (BCS, log*β*_2_ = 20.8)^48^ was mainly used to buffer free Cu(I). An association constant (n = 5) of (1.7 ± 0.5) × 10^17^ M^−1^ was obtained for *Mt*Csp3 with a Hill coefficient of 1.0 ± 0.1 (Fig. 3c,d). The calculated occupancies of 16.2 to 16.9 per monomer for the *Mt*Csp3 samples from Fig. 3c incubated with 36.6 to 39.0 equivalents of Cu(I) for 67 h (see Supplementary Fig. S9 for the stability of *Mt*Csp3 over time) were checked. To do this the protein was separated from [Cu(BCS)_2_]^3−^ and free BCS, and an *Mt*Csp3 monomer was found to bind 20.9 equivalents of Cu(I). Using BCA as the Cu(I)-buffering ligand (n = 1), a Cu(I) affinity (association constant) of (2.0 ± 0.1) × 10^17^ M^−1^ (Hill coefficient of 1.3 ± 0.1) was obtained (86 h incubation), but the maximum calculated occupancy was low (~11), although an *Mt*Csp3 monomer bound 16.0 equivalents of Cu(I) when separated from [Cu(BCA)_2_]^3−^ and free BCA (see Supplementary Methods). For *Bs*Csp3 (n = 3) the average Cu(I) affinity (association constant) is (1.5 ± 0.4) × 10^17^ M^−1^, with a Hill coefficient of 0.9 ± 0.1 (Fig. 3e,f). The calculated occupancies of 17.8 to 18.6 equivalents for samples from Fig. 3e to which 36.6 to 39.5 equivalents of Cu(I) had been added (after 138 h incubation, see Supplementary Fig. S9 for the stability of *Bs*Csp3 over time), were found to bind 20.2 equivalents of Cu(I) per monomer after separation from [Cu(BCS)_2_]^3−^ and free BCS.

### Cu(I) removal from Csp3s

**Figure 5:**
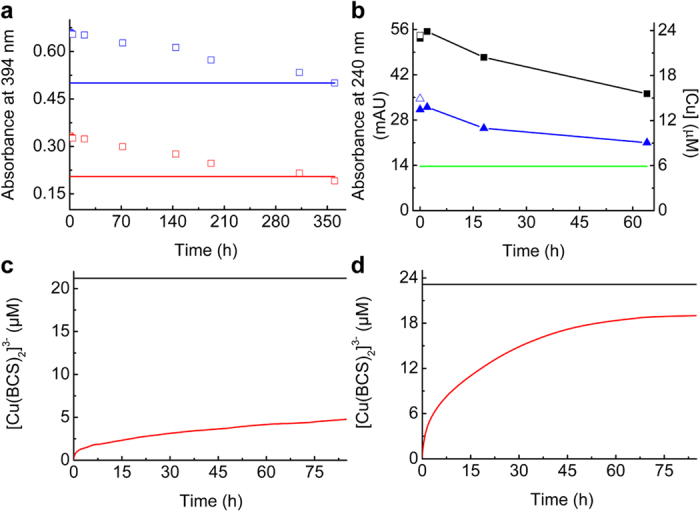
Slow Cu(I) removal from the Csp3s. (**a**) Plots of the absorbance at 394 nm against time (UV-Vis spectra shown in Supplementary Fig. S10) after the addition of *Mt*Csp3 (0.96 and 0.94 μM respectively) loaded with 18.0 equivalents of Cu(I) to apo-mbtin (17.1 μM, open red squares and 33.9 μM, open blue squares). Also shown are the final absorbance values upon the addition of Cu(I) only (16.7 μM) to apo-mbtin at 16.8 (red line) and 33.8 (blue line) μM. (**b**) Gel-filtration analysis of *Bs*Cps3 (3.40 μM) plus 15.1 equivalents of Cu(I) mixed with apo-*Bs*CopZ (100 μM). The maximum absorbance at 240 nm for eluted *Bs*Csp3 (filled black squares), the amount of copper associated with this peak (filled blue triangles) and the absorbance at 240 nm of apo-*Bs*Csp3 (green line) are shown. The open symbols are the absorbance at 240 nm (black square) and copper content (blue triangle) for Cu(I)-*Bs*Csp3 at the same concentration (3.40 μM). Plots (red lines) of Cu(I) removal by BCS (2.41 mM) from *Mt*Csp3 (1.26 μM, **c**) and *Bs*Csp3 (1.24 μM, **d**) loaded with 16.6 and 18.0 equivalents of Cu(I) respectively (for the stability of *Mt*Csp3 and *Bs*Csp3 over time see Supplementary Fig. S9). The black lines indicate the [Cu(BCS)_2_]^3−^ concentration of the same Csp3 samples after 2 h in the presence of 6.5 M guanidine hydrochloride (Supplementary Fig. S10). All experiments were performed in 20 mM Hepes plus 200 mM NaCl at pH 7.5 (500 mM NaCl for the gel-filtration analyses in **b**).

Mbtin from *M. trichosporium* OB3b has a very high Cu(I) affinity^27^,^48^ of (6–7) × 10^21^ M^−1^ at pH 7.5 and removes all Cu(I) from *Mt*Csp3 loaded with 18.0 Cu(I) equivalents (Fig. 5a), but this takes approximately 15 days. Cu(I) removal from Cu(I)-*Bs*Csp3 by apo-*Bs*CopZ, which has a Cu(I) affinity^8^,^9^,^48^ of ~10^18^ M^−1^, has also been analyzed. After 64 h (Fig. 5b) ~35–45% of Cu(I) transfers from *Bs*Csp3 to *Bs*CopZ (see Supplementary Methods). Thus Cu(I) removal by possible physiological partners is very slow for both Csp3s. The ability of Csp3s to hold onto Cu(I) was further investigated using BCS, with a very large excess removing only ~20% of Cu(I) from *Mt*Csp3 after 85 h (Fig. 5c), whilst ~85% removal from *Bs*Csp3 is observed over the same timescale (Fig. 5d).

### *In vivo* studies of *Bs*Csp3

**Figure 6:**
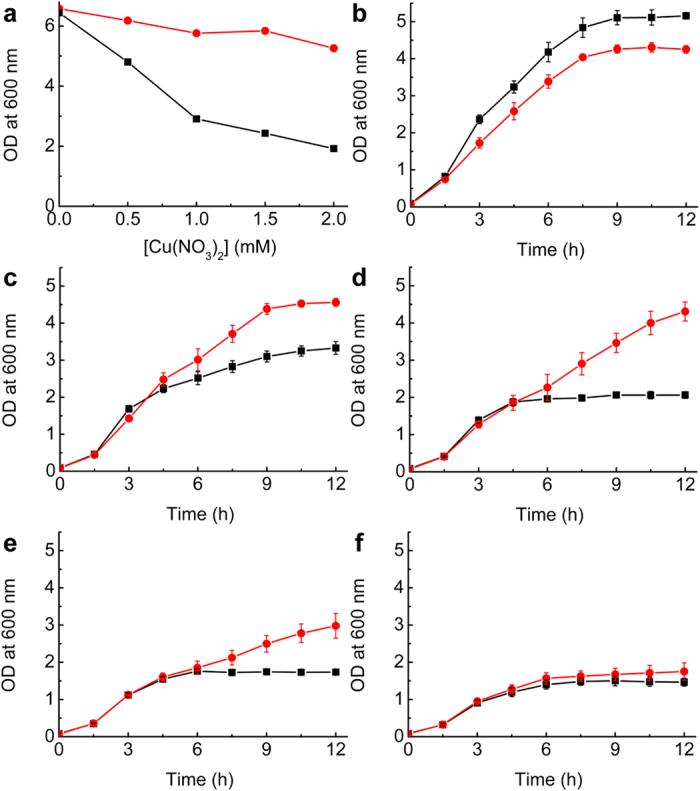
*In vivo* studies of *Bs*Csp3. (**a**) Growth at 37 °C of WT *E. coli* BW25113 (red circles) and the Δ*copA* strain (black squares) after 6 h in LB media plus increasing concentrations of copper nitrate. Also shown are growth curves at 37 °C of Δ*copA* (black squares) and the same strain plus pBAD33_*Bs*Csp3 (red circles) at 0 (**b**), 0.5 (**c**), 1.0 (**d**), 1.5 (**e**), and 2.0 (**f**) mM copper nitrate. The averages and standard deviations from three independent growth experiments are shown in (**b** to **f**), with the data for Δ*copA* including two experiments plus pBAD33. Δ*copA* plus pBAD33_*Bs*Csp3 tested after 12 h growth in media plus 0.5, 1.0 and 1.5 mM copper nitrate contains approximately two times more copper than control cells (Δ*copA* plus pBAD33) grown under the same conditions.

To test the ability of Csp3 to provide protection against copper toxicity, *Bs*Csp3 was introduced into a Δ*copA E. coli* strain, which exhibited diminished growth upon increasing copper concentration in the media compared to wild type (WT) (Fig. 6a)^53^. The expression of *Bs*Csp3 reverses this effect allowing Δ*copA E. coli* to grow for significantly longer than WT, particularly at 0.5 to 1.5 mM copper (Fig. 6b–f). Furthermore, cells grown under these conditions expressing *Bs*Csp3 accumulate approximately two times more copper than Δ*copA* control cells. An initial analysis of the Δ*csp3* strain of *B. subtilis* has also been undertaken (see Supplementary Methods), which exhibits enhanced cell death compared to WT in the presence of 1.5 to 2.0 mM copper (Supplementary Fig. S11).

## Discussion

The Csp3s are the cytosolic members of a new family of copper storage proteins^22^. Both the *M. trichosporium* OB3b and *B. subtilis* proteins form tetramers of four-helix bundles (Fig. 1) possessing many Cys residues pointing into their cores, and can accommodate up to approximately 80 Cu(I) ions per tetramer. Csp3s are present in many other bacteria (Supplementary Fig. S1) and presumably have similar structures and Cu(I)-binding capabilities to the proteins studied here. The Cu(I) capacity of the Csp3s is much greater than that of a *Mt*Csp1 tetramer, which binds a maximum of 52 Cu(I) ions^22^. The number of Cu(I) ions a Csp can bind is dictated by how many Cys residues it possesses, with 18 and 19 respectively per monomer in *Mt*Csp3 and *Bs*Csp3 compared to 13 in *Mt*Csp1. Copper coordination in *Mt*Csp3 largely involves sites with two Cys ligands, with the most novel being Cu13, which as well as having two coordinating thiolates is bound by the side chain amide oxygen of Asn58 (Fig. 4c). This Asn is not highly conserved in Csp3s (Supplementary Fig. S1), and an Asp is more common, as found in *Bs*Csp3 (Fig. 2b). The high density of Cys residues within the core of the Csp3s and the absence of facile disulfide bond formation is remarkable. The Cys residues all originate from α-helices and the four-helix bundle scaffold provides the rigidity required to hold these residues sufficiently far apart to prevent thiol groups coupling via oxidation (in a cell this will be assisted by the reducing environment of the cytosol). This is essential as disulfide formation would hinder, and perhaps even prevent, Cu(I) binding.

The lack of cooperativity of Cu(I) binding by the Csp3s highlights that their Cu(I)-uptake mechanism probably differs compared to that of *Mt*Csp1 (the Hill coefficient is close to 1 for both Csp3s, whereas for *Mt*Csp1 it is ~3, indicating positive cooperativity). This is consistent with luminescence at around 600 nm for Csp3s upon Cu(I) addition (Supplementary Figs S6 and S7), whereas very little emission is observed for *Mt*Csp1^22^. The average Cu(I) affinities of *Mt*Csp3, *Bs*Csp3 and *Mt*Csp1 are all in the (1–2) × 10^17^ M^−1^ range. The slow removal of Cu(I) by a range of partners, all with tighter Cu(I) affinities, is a highly unusual feature of the Csp3s compared to the exported *Mt*Csp1^22^ and MT^54^ (the eukaryotic cytosolic Cu(I) storage protein), which can both be very quickly stripped of all Cu(I). Removal of iron from the mineral core of ferritin can also occur on a similarly short timescale^1^,^55^. A comparison of Cu(I)-removal rates for *Mt*Csp3, *Bs*Csp3 and *Mt*Csp1 is obtained using BCS. Cu(I) removal by this high affinity small molecule ligand is slowest for *Mt*Csp3, faster for *Bs*Csp3 (Fig. 5c,d), but is orders of magnitude quicker for *Mt*Csp1, with all Cu(I) removed in ~30 min^22^.

One end of a Csp four-helix bundle is hydrophobic whilst at the opposite end the Cu(I) sites are more solvent exposed (Fig. 2), which presumably is the route via which Cu(I) ions enter and leave. Structural variations between the Csp3s and *Mt*Csp1 at this opening could be responsible for the dramatically different rates of Cu(I)-removal. His104, His108 and His110 (His104 and His110 are ligands, Fig. 4c) from the α3-loop-α4 region are found around the opening to the bundle in *Mt*Csp3 (Figs 1a and 2a) and are highly conserved among Csp3s (Supplementary Fig. S1), corresponding to His84, His86 and His88 in *Bs*Csp3 (Figs 1b and 2b). At the entrance of the *Mt*Csp1 bundle (Fig. 2c)^22^ there is only a single His residue (His36 that coordinates Cu13) and three largely conserved methionines; Met40, Met43 and Met48 (the latter is a bridging ligand between Cu11 and Cu13), on α1 and the loop linking α1 with α2. Leu51 of *Mt*Csp3 (Fig. 2a), which is conserved in *Bs*Csp3 (Leu35, Fig. 2b), corresponds to Met48 in *Mt*Csp1 (Fig. 2c) and its hydrophobic side chain appears to partially block the entrance (a Met is actually found in this position in a number of Csp3s, Supplementary Fig. S1). The residues at the opening of the bundle could influence Cu(I)-removal rates from the cores of these proteins.

Physiologically, *Mt*Csp3 could acquire copper imported either via the mbtin-dependent uptake system or from CopD. *Bs*Csp3 may receive copper via YcnJ, which has homology to CopD. It is possible that Cu(I) removal from *Mt*Csp3 is carried out by apo-mbtin when copper availability decreases in *M. trichosporium* OB3b, although *in vitro* this process occurs very slowly. *B. subtilis* is not known to possess a high affinity Cu(I) ligand, but it does produce bacillithiol^56^. However, this small molecule antioxidant has been implicated in Zn(II) buffering^57^. Cu(I) acquisition from *Bs*Csp3 by apo-*Bs*CopZ, the cytosolic copper metallochaperone not present in *M. trichosporium* OB3b, is also slow *in vitro*. In *B. subtilis* and *M. trichosporium* OB3b Cu(I) could be passed from Csp3 to a metal-binding domain of CopA that has a very similar structure to CopZ^47^,^52^, or to the copper-dependent transcriptional activators, CueR and CsoR respectively. However, these all bind Cu(I) in a similar manner to CopZ^15^, and therefore transfer is also expected to be slow. Once bound by Csp3, Cu(I) ions appear to be trapped, which would be expected to help ensure non-specific removal in the cytosol preventing toxicity. This is consistent with the ability of *Bs*Csp3 to allow the Δ*copA E. coli* strain to grow for significantly longer than WT at copper concentrations above 0.5 mM (Fig. 6c–e) and to accumulate intracellular copper. Furthermore, Δ*csp3 B. subtilis* exhibits enhanced cell death above 1.0 mM copper compared to WT (Supplementary Fig. S11). The Csp3s can bind copper in the cytosol of bacteria conferring the ability to grow at elevated levels of this metal.

Slow removal of Cu(I) from Csp3s observed *in vitro* implies that either these proteins are involved in long-term copper storage or that mechanisms are present in cells to facilitate the delivery of their metal cargo to specific targets. *Mt*Csp3 could act as a longer-term store of copper for pMMO in *M. trichosporium* OB3b, compared to more immediate supply^22^ by *Mt*Csp1 and *Mt*Csp2. Transcriptomic studies have shown that the *csp3* gene is up-regulated in *B. subtilis* under various stress-inducing/spore-forming conditions^58^. One of many outer spore coat proteins is CotA, a laccase belonging to the multi-copper oxidase family^43^, and *Bs*Csp3 may store copper for this enzyme. Furthermore, in both organisms Csp3-bound copper potentially supplies currently unknown copper enzymes. This is consistent with the number of high-abundance copper fractions that are resolved from soluble extracts of *M. trichosporium* OB3b^22^. Some Csp3-containing bacteria are pathogenic and Csp3 could be important in allowing these to respond to copper-related attack by a host’s immune system^16^. In conclusion, the presence of Csp3 will allow a bacterium to maintain a safe, and potentially large, cytosolic source of copper, and work is currently underway to identify physiological targets for Csp3-bound Cu(I).

## Materials and Methods

### Protein production and analysis

The methods used to clone, over-express, purify, quantify and analyze the Csp3s and *Bs*CopZ are similar to those described previously^22^,^59^, and are given in detail in the Supplementary Methods. Protein concentrations were determined using the DTNB assay, with unfolded apo-proteins when quantifying Csp3s, under anaerobic conditions^22^,^47^. Csp3 concentrations were also determined with the Bradford assay. Proteins were analyzed using atomic absorption spectrometry (AAS), far-UV (180–250 nm) CD spectroscopy and analytical gel-filtration chromatography^9^,^10^,^22^,^47^,^60^,^61^.

### Preparation of Cu(I) samples, Cu(I)-binding stoichiometry, estimated average Cu(I) affinities and Cu(I) removal

Most experiments were performed in 20 mM Hepes plus 200 mM NaCl at pH 7.5 and are described in detail in the Supplementary Methods. Work with Cu(I) and Cu(I)-proteins was performed under anaerobic conditions^22^,^47^. To prepare Cu(I)-Csp3 samples, the appropriate amount of a buffered Cu(I) solution (made from a Cu(I) stock in acetonitrile) was mixed with apo-protein quantified by DTNB in the presence of either urea or guanidine hydrochloride, with Cu(I) equivalents per monomer quoted in all cases. Direct Cu(I) titrations into apo-Csp3s were monitored by UV-Vis and fluorescence spectrophotometry. To determine the number of tightly bound equivalents per monomer, Cu(I) was added to apo-Csp3 in the presence of ~40 equivalents of BCA and the formation of [Cu(BCA)_2_]^3−^ quantified by UV-Vis spectrophotometry^22^,^60^. Average Cu(I) affinities were measured mainly using BCS and an approach described previously^22^,^54^. For these measurements mixtures of Csp3 (~2.5 μM, 2.09 μM on one occasion for *Bs*Csp3) and BCS (100–121 μM) plus increasing concentrations of Cu(I) were incubated anaerobically for up to 124 h for *Mt*Csp3 and 138 h for *Bs*Csp3, with reported affinities from data measured after 42–43 h and 90–96 h respectively (no significant change observed at the longer incubation times). The ability of *M. trichosporium* OB3b mbtin to remove Cu(I) from *Mt*Csp3, and Cu(I) removal from *Mt*Csp3 and *Bs*Csp3 by BCS, were both monitored by UV-Vis spectrophotometry^22^. Cu(I) removal from Cu(I)-*Bs*Csp3 by apo-*Bs*CopZ was followed by analyzing an anaerobic mixture of the proteins using gel-filtration chromatography. Many of the above experiments required Csp3 samples to be incubated over many days and the stability of proteins over the same time period was tested by far-UV CD spectroscopy.

### *In vivo* studies using the *copA* deletion strain of *Escherichia coli*

The published^53^ copper-dependent phenotype for a Δ*copA E. coli* was confirmed as described in the Supplementary Methods. *Bs*Csp3 was sub-cloned from pET29a into the *Xba*I and *Hind*III sites of pBAD33 giving pBAD33_*Bs*Csp3. The growth of Δ*copA* transformed with pBAD33_*Bs*Csp3 was investigated at a range of copper concentrations (details in the Supplementary Methods). Copper levels in cells were measured by AAS as described in the Supplementary Methods.

### Crystallization, data collection, structure solution and refinement

Diffraction-quality crystals were obtained as described in the Supplementary Methods. All crystallographic data were collected at Diamond Light Source Ltd, UK (beamline I02) and structures were solved by molecular replacement using Molrep implemented via the CCP4 suite^62^. Models underwent building cycles in Coot^63^ and refinement in REFMAC5^64^. Five percent of observations were used to monitor refinement. All models were validated using Molprobity^65^ and data collection statistics and refinement details are reported in Supplementary Table S1. Atomic coordinates have been deposited in the Protein Data Bank under accession numbers 5ARM, 5ARN and 5FIG for apo-*Mt*Csp3, Cu(I)-*Mt*Csp3 and apo-*Bs*Csp3 respectively.

## Additional Information

**How to cite this article**: Vita, N. *et al*. Bacterial cytosolic proteins with a high capacity for Cu(I) that protect against copper toxicity. *Sci. Rep.* **6**, 39065; doi: 10.1038/srep39065 (2016).

**Publisher's note:** Springer Nature remains neutral with regard to jurisdictional claims in published maps and institutional affiliations.

## Supplementary Material

Supplementary Information

## References

[b1] Theil E. C. Ferritin protein nanocages use ion channels, catalytic sites, and nucleation channels to manage iron/oxygen chemistry. Curr. Opin. Chem. Biol. 15, 304–311 (2011). 21296609 10.1016/j.cbpa.2011.01.004 PMC3074017

[b2] Sutherland D. E. K. & Stillman M. J. The “magic numbers” of metallothionein. Metallomics 3, 444–463 (2011). 21409206 10.1039/c0mt00102c

[b3] Macomber L. & Imlay J. A. The iron-sulfur clusters of dehydratases are primary intracellular targets of copper toxicity. Proc. Natl. Acad. Sci. USA 106, 8344–8349 (2009). 19416816 10.1073/pnas.0812808106 PMC2688863

[b4] Kim B. E., Nevitt T. & Thiele D. J. Mechanisms for copper acquisition, distribution and regulation. Nat. Chem. Biol. 4, 176–185 (2008). 18277979 10.1038/nchembio.72

[b5] Festa R. A. & Thiele D. J. Copper: an essential metal in biology. Curr. Biol. 21, R877–R883 (2011). 22075424 10.1016/j.cub.2011.09.040 PMC3718004

[b6] Argüello J. M., Raimunda D. & Padilla-Benavides T. Mechanisms of copper homeostasis in bacteria. Front. Cell. Infect. Microbiol. 3, 73 (2013). 24205499 10.3389/fcimb.2013.00073 PMC3817396

[b7] Gourdon P. . Crystal structure of a copper-transporting PIB-type ATPase. Nature 475, 59–64 (2011). 21716286 10.1038/nature10191

[b8] Zhou L., Singleton C. & Le Brun N. E. High Cu(I) and low proton affinities of the CXXC motif of *Bacillus subtilis* CopZ. Biochem. J. 413, 459–465 (2008). 18419582 10.1042/BJ20080467

[b9] Badarau A. & Dennison C. Copper trafficking mechanism of CXXC-containing domains: insight from the pH-dependence of their Cu(I) affinities. J. Am. Chem. Soc. 133, 2983–2988 (2011). 21323310 10.1021/ja1091547

[b10] Badarau A. & Dennison C. Thermodynamics of copper and zinc distribution in the cyanobacterium *Synechocystis* PCC 6803. Proc. Natl. Acad. Sci. USA 108, 13007–13012 (2011). 21778408 10.1073/pnas.1101448108 PMC3156197

[b11] Xiao Z. . Unification of the copper(I) binding affinities of the metallo-chaperones Atx1, Atox1, and related proteins. J. Biol. Chem. 286, 11047–11055 (2011). 21258123 10.1074/jbc.M110.213074 PMC3064159

[b12] Poutney D. L., Schauwecker I., Zarn J. & Vašák M. Formation of mammalian Cu_8_-metallothionein *in vitro*: evidence for the existence of two Cu(I)_4_-thiolate clusters. Biochemistry 33, 9699–9705 (1994). 8068648 10.1021/bi00198a040

[b13] Calderone V. . The crystal structure of yeast copper thionein: the solution of a long-lasting enigma. Proc. Natl. Acad. Sci. USA 102, 51–56 (2005). 15613489 10.1073/pnas.0408254101 PMC544076

[b14] Gold B. . Identification of a copper-binding metallothionein in pathogenic bacteria. Nat. Chem. Biol. 4, 609–616 (2008). 18724363 10.1038/nchembio.109 PMC2749609

[b15] Davis A. V. & O’Halloran T. V. A place for thioether chemistry in cellular copper ion recognition and trafficking. Nat. Chem. Biol. 4, 148–151 (2008). 18277969 10.1038/nchembio0308-148 PMC2265432

[b16] Fu Y., Chang F. M. J. & Giedroc D. P. Copper transport and trafficking at the host-bacterial pathogen interface. Acc. Chem. Res. 47, 3605–3613 (2014). 25310275 10.1021/ar500300n PMC4268108

[b17] Balasubramanian R. . Oxidation of methane by a biological dicopper centre. Nature 465, 115–119 (2010). 20410881 10.1038/nature08992 PMC2999467

[b18] DiSpirito A. A. . Methanobactin and the link between copper and bacterial methane oxidation. Microbiol. Mol. Biol. Rev. 80, 387–409 (2016). 26984926 10.1128/MMBR.00058-15 PMC4867365

[b19] Jiang H. . Methanotrophs: multifunctional bacteria with promising applications in environmental bioengineering. Biochem. Eng. J. 49, 277–288 (2010).

[b20] Haynes C. A. & Gonzalez R. Rethinking biological activation of methane and conversion to liquid fuels. Nat. Chem. Biol. 10, 331–339 (2014). 24743257 10.1038/nchembio.1509

[b21] Strong P. J., Kalyuzhnaya M., Silverman J. & Clarke W. P. A methanotroph-based biorefinery: potential scenarios for generating multiple products from a single fermentation. Bioresource Technol. 215, 314–323 (2016). 10.1016/j.biortech.2016.04.099 27146469

[b22] Vita N. . A four-helix bundle stores copper for methane oxidation. Nature 525, 140–143 (2015). 26308900 10.1038/nature14854 PMC4561512

[b23] Davies S. L. & Whittenbury R. Fine structure of methane and other hydrocarbon-utilising bacteria. J. Gen. Microbiol. 61, 227–232 (1970). 5476893 10.1099/00221287-61-2-227

[b24] Changela A. . Molecular basis of metal-ion selectivity and zeptomolar sensitivity by CueR. Science 301, 1383–1387 (2003). 12958362 10.1126/science.1085950

[b25] Stein L. Y. . Genome sequence of the obligate methanotroph *Methylocystis trichosporium* strain OB3b. J. Bacteriol. 192, 6497–6498 (2010). 20952571 10.1128/JB.01144-10 PMC3008524

[b26] Kim H. J. . Methanobactin, a copper-acquisition compound from methane-oxidizing bacteria. Science 305, 1612–1615 (2004). 15361623 10.1126/science.1098322

[b27] El Ghazouani A. . Copper-binding properties and structures of methanobactins from *Methylosinus trichosporium* OB3b. Inorg. Chem. 50, 1378–1391 (2011). 21254756 10.1021/ic101965j

[b28] Kenny G. E. & Rosenzweig A. C. Chemistry and biology of the copper chelator methanobactin. ACS Chem. Biol. 7, 260–268 (2012). 22126187 10.1021/cb2003913 PMC3288317

[b29] El Ghazouani A. . Variations in methanobactin structure influences copper utilization by methane-oxidizing bacteria. Proc. Natl. Acad. Sci. USA 109, 8400–8404 (2012). 22582172 10.1073/pnas.1112921109 PMC3365152

[b30] Gu W. . A Ton-B-dependent transporter is responsible for methanobactin uptake by *Methylosinus trichosporium* OB3b. Appl. Environ. Microbiol. 82, 1917–1923 (2016). 26773085 10.1128/AEM.03884-15 PMC4784032

[b31] Balasubramanian R., Kenny G. E. & Rosenzweig A. C. Dual pathways for copper uptake by methanotrophic bacteria. J. Biol. Chem. 286, 37313–37319 (2011). 21900235 10.1074/jbc.M111.284984 PMC3199478

[b32] Kenney G. E., Sadek M. & Rosenzweig A. C. Copper-responsive gene expression in the methanotroph *Methylosinus trichosporium* OB3b. Metallomics 8, 931–940 (2016). 27087171 10.1039/c5mt00289c PMC6195801

[b33] Moore C. M. & Helmann J. D. Metal ion homeostasis in *Bacillus subtilis*. Curr. Opin. Microbiol. 8, 188–195 (2005). 15802251 10.1016/j.mib.2005.02.007

[b34] Solioz M., Abicht H. K., Mermod M. & Mancini S. Response of Gram-positive bacteria to copper stress. J. Biol. Inorg. Chem. 15, 3–14 (2010). 19774401 10.1007/s00775-009-0588-3

[b35] Helmann J. D. Specificity of metal sensing: Iron and manganese homeostasis in *Bacillus subtilis*. J. Biol. Chem. 289, 28112–28120 (2014). 25160631 10.1074/jbc.R114.587071 PMC4192466

[b36] Smaldone G. T. & Helmann J. D. CsoR regulates the copper efflux operon *copZA* in *Bacillus subtilis*. Microbiology 153, 4123–4128 (2007). 18048925 10.1099/mic.0.2007/011742-0 PMC3019219

[b37] Liu T. . CsoR is a novel *Mycobacterium tuberculosis* copper-sensing transcriptional regulator. Nat. Chem. Biol. 3, 60–68 (2007). 17143269 10.1038/nchembio844

[b38] Ma Z., Cowart D. M., Scott R. A. & Giedroc D. P. Molecular insight into the metal selectivity of the copper(I)-sensing repressor CsoR from *Bacillus subtilis*. Biochemistry 48, 3325–3334 (2009). 19249860 10.1021/bi900115w PMC2728441

[b39] Gaballa A. & Helmann J. D. *Bacillus subtilis* CPx-type ATPases: Characterization of Cd, Zn, Co and Cu efflux systems. BioMetals 16, 497–505 (2003). 12779235 10.1023/a:1023425321617

[b40] Radford D. S. . CopZ from *Bacillus subtilis* interacts *in vivo* with a copper exporting CPx-type ATPase CopA. FEMS Microbiol. Lett. 220, 105–112 (2003). 12644235 10.1016/S0378-1097(03)00095-8

[b41] Sarasta M. . The *Bacillus subtilis* cytochrome-*c* oxidase. Eur. J. Biochem. 195, 517–525 (1991). 1847686 10.1111/j.1432-1033.1991.tb15732.x

[b42] Lauraeus M., Haltia T., Saraste M. & Wikström M. *Bacillus subtilis* expresses two kinds of haem-A-containing terminal oxidases. Eur. J. Biochem. 197, 699–705 (1991). 1851483 10.1111/j.1432-1033.1991.tb15961.x

[b43] Hullo M. F., Moszer I., Danchin A. & Martin-Verstraete I. CotA of *Bacillus subtilis* is a copper-dependent laccase. J Bacteriol. 183, 5426–5430 (2001). 11514528 10.1128/JB.183.18.5426-5430.2001 PMC95427

[b44] Ridge P. G., Zhang Y. & Gladyshev V. N. Comparative genomic analyses of copper transporters and cuproproteomes reveal evolutionary dynamics of copper utilization and its link to oxygen. PLoS One 3, e1378 (2008). 18167539 10.1371/journal.pone.0001378 PMC2147054

[b45] Banci L. . A prokaryotic superoxide dismutase paralog lacking two Cu ligands: from largely unstructured in solution to ordered in the crystal. Proc. Natl. Acad. Sci. USA 102, 7541–7546 (2005). 15897454 10.1073/pnas.0502450102 PMC1140445

[b46] Chillappagari S., Miethke M., Trip H., Kuipers O. P. & Marahiel M. M. Copper acquisition is mediated by YcnJ and regulated by YcnK and CsoR in *Bacillus subtilis*. J. Bacteriol. 191, 2362–2370 (2009). 19168619 10.1128/JB.01616-08 PMC2655523

[b47] Badarau A., Firbank S. J., McCarthy A. A., Banfield M. J. & Dennison C. Visualizing the metal-binding versatility of copper trafficking sites. Biochemistry 49, 7798–7810 (2010). 20726513 10.1021/bi101064w

[b48] Bagchi P., Morgan M. T., Bacsa J. & Fahrni C. J. Robust Affinity Standards for Cu(I) Biochemistry. J. Am. Chem. Soc. 135, 18549–18559 (2013). 24298878 10.1021/ja408827d PMC3983694

[b49] Green A. R., Presta A., Gansyna Z. & Stillman M. J. Luminescent probe of copper-thiolate cluster formation within mammalian metallothionein. Inorg. Chem. 33, 4159–4168 (1994).

[b50] Chen X. . Copper sensing function of *Drosophila* metal-responsive transcriptional factor-1 is mediated by a tetranuclear Cu(I) cluster. Nucleic Acid Res. 36, 3128–3138 (2008). 18411209 10.1093/nar/gkn103 PMC2396432

[b51] Xie F., Sutherland D. E. K., Stillman N. J. & Ogawa M. Y. Cu(I) binding properties of a designed metalloprotein. J. Inorg. Biochem. 104, 261–267 (2010). 20060593 10.1016/j.jinorgbio.2009.12.005

[b52] Boal A. K. & Rosenzweig A. C. Structural biology of copper trafficking. Chem. Rev. 109, 4760–4779 (2009). 19824702 10.1021/cr900104z PMC2768115

[b53] Rensing C., Fan B., Sharma R., Mitra B. & Rosen B. P. CopA: an *Escherichia coli* Cu(I)-translocating P-type ATPase. Proc. Natl. Acad. Sci. USA 97, 652–656 (2000). 10639134 10.1073/pnas.97.2.652 PMC15385

[b54] Banci L. . Affinity gradients drive copper to cellular destinations. Nature 465, 645–648 (2010). 20463663 10.1038/nature09018

[b55] Yasmin S., Andrews S. C., Moore G. R. & Le Brun N. E. A new role for heme, facilitating release of iron from the bacterioferritin iron mineral. J. Biol. Chem. 286, 3473–3483 (2011). 21106523 10.1074/jbc.M110.175034 PMC3030353

[b56] Newton G. L. . Bacillithiol is an antioxidant thiol produced in Bacilli. Nat. Chem. Biol. 5, 625–627 (2009). 19578333 10.1038/nchembio.189 PMC3510479

[b57] Ma Z. . Bacillithiol is a major buffer of the labile zinc pool in *Bacillus subtilis*. Mol. Micro. 94, 756–770 (2014). 10.1111/mmi.12794 PMC4227968 25213752

[b58] Nicolas P. . Condition-dependent transcriptome reveals high-level regulatory architecture in *Bacillus subtilis*. Science 335, 1103–1106 (2012). 22383849 10.1126/science.1206848

[b59] Kihlken M. A., Leech A. P. & Le Brun N. E. Copper-mediated dimerization of CopZ, a predicted copper chaperone from *Bacillus subtilis*. Biochem. J. 368, 729–739 (2002). 12238948 10.1042/BJ20021036 PMC1223043

[b60] Allen S., Badarau A. & Dennison C. Cu(I) affinities of the domain 1 and 3 sites in the human metallochaperone for Cu, Zn-superoxide dismutase. Biochemistry 51, 1439–1448 (2012). 22320662 10.1021/bi201370r

[b61] Allen S., Badarau A. & Dennison C. The influence of protein folding on the copper affinities of trafficking and target sites. Dalton Trans. 42, 3233–3239 (2013). 23169585 10.1039/c2dt32166a

[b62] Winn M. D. . Overview of the CCP4 suite and current developments. Acta. Cryst. D67, 235–242 (2011). 10.1107/S0907444910045749 PMC3069738 21460441

[b63] Emsley P., Lohkamp B., Scott W. G. & Cowtan K. Features and development of Coot. Acta Cryst. D66, 486–501 (2010). 10.1107/S0907444910007493 PMC2852313 20383002

[b64] Vagin A. A. . REFMAC5 dictionary: organisation of prior chemical knowledge and guidelines for its use. Acta Cryst. D60, 2284–2295 (2004). 10.1107/S0907444904023510 15572771

[b65] Chen V. B. . MolProbity: all-atom structure validation for macromolecular crystallography. Acta Cryst. D66 (Pt 1), 12–21 (2010). 10.1107/S0907444909042073 PMC2803126 20057044

