# Supplementary material for: Bacterial cytosolic proteins with a high capacity for Cu(I) that protect against copper toxicity
Source: Sci Rep. 2016 Dec 19;6:39065. doi: 10.1038/srep39065 (PMC5171941; doi:10.1038/srep39065)
Supplement: Supplementary Information [file srep39065-s1.pdf]

Supplementary Information

Bacterial cytosolic proteins with a high capacity for Cu(I) that protect against copper toxicity

Nicolas Vita, Gianpiero Landolfi, Arnaud Baslé, Semeli Platsaki, Jaeick Lee, Kevin J. Waldron, and Christopher Dennison

Institute for Cell and Molecular Biosciences, Medical School, Newcastle University, Newcastle upon Tyne NE2 4HH, UK

Supplementary Methods

Cloning the *csp3* gene. The *Methylosinus trichosporium* OB3b *csp3* gene was amplified from genomic DNA using the primers 5'-GCG**CATATG**CATGTGGAAGCC-3' (forward, *Nde*I restriction site in bold) and 5'-GCG**CCATGG**<u>CTA</u>ATGCGTGAGCCCCGTCGC-3' (reverse, *Nco*I restriction site in bold, stop codon underlined). The *csp3* gene (*yhjQ*) from *Bacillus subtilis* 168 was amplified from genomic DNA using the primers 5'-GCG**CATATG**GAGCAATATTCTGAGGC-3' (forward, *Nde*I restriction site in bold) and 5'GCG**CCATGG**<u>TTA</u>CGCTGCCATGCTGCGGC-3' (reverse, *Nco*I restriction site in bold, stop codon underlined). Fragments were cloned into pGEM-T, verified by sequencing, and sub-cloned into the *Nde*I and *Nco*I sites of pET29a to give pET29a-*Mt*Csp3 and pET29a-*Bs*Csp3 respectively.

Expression and purification of the Csp3 proteins. *Escherichia coli* BL21 (DE3) transformed with pET29a-*Mt*Csp3 was grown in LB media at 37 ºC (100 μg/ml kanamycin) until an OD_{600} of ~0.6 when cells were induced with either 0.1 or 1 mM isopropyl β-D-thiogalactopyranoside (IPTG), harvested after 24 h and stored at -30 ºC. The protein was purified as described previously for *Mt*Csp1[1], except that *Mt*Csp3 fractions identified by sodium dodecyl sulfate polyacrylamide gel electrophoresis (SDS-PAGE) from the first anion-exchange column were combined, diluted 10-fold in 10 mM tris(hydroxymethyl)aminomethane (Tris) pH 7.0 plus 1 mM dithiothreitol (DTT), and applied to either a HiTrap Q FF or a HP anion-exchange column (5 ml, GE Healthcare) equilibrated in the same buffer and eluted using a linear NaCl gradient (0-200 mM, total volume ~200 ml). Essentially pure *Mt*Csp3 could be obtained from this column and was used for crystallisation samples and some *in vitro* experiments. The protein at this stage can contain a very small amount of a heme-containing protein and further purification was achieved on a Superdex 75 10/300 GL column (GE Healthcare) as described previously[1]. Purified *Mt*Csp3 contained no copper or zinc determined (n = 6) by atomic absorption spectroscopy (AAS, see below), and has a mass of 14524.6 Da determined by Fourier transform ion cyclotron resonance mass spectrometry (FT-ICR-MS), consistent with the recombinant protein having Met1 and His133 at its N- and C-termini respectively (calculated mass 14524.7 Da).

*Bs*Csp3 was over-expressed in *E. coli* BL21 (DE3) transformed with pET29a-*Bs*Csp3 as described above for *Mt*Csp3, using 0.1 mM IPTG for induction. Pellets were resuspended in 20 mM Tris pH 8.0, sonicated, and centrifuged at 40000 g for 25 min. The supernatant was diluted 5-fold in 20 mM Tris pH 8.0, loaded onto two connected HiTrap Q FF anion-exchange columns (5 ml) equilibrated in the same buffer, and proteins eluted with a linear NaCl gradient (0 to 400 mM, total volume ~240 ml). The *Bs*Csp3-containing fractions were identified by SDS-PAGE, combined, diluted 6 to 8-fold in 20 mM Tris pH 8.0 and loaded onto two connected HiTrap Q FF columns (5

ml). Proteins were eluted using a linear NaCl gradient (0 to 300 mM, ~240 ml) in 20 mM 2-(N-morpholino)ethanesulfonic acid (Mes) pH 6.5. The purest fractions, identified by SDS-PAGE, were combined, diluted 10-fold in 20 mM 4-(2-hydroxyethyl)piperazine-1-ethanesulfonic acid (Hepes) pH 7.5 and loaded onto a HiTrap Q HP column (1 ml), equilibrated in the same buffer. *Bs*Csp3 was eluted with 20 mM Hepes pH 7.5 plus 500 mM NaCl and further purified by gel-filtration chromatography on a Superdex 75 10/300 GL column equilibrated in the same buffer. Fractions containing *Bs*Csp3, identified by SDS-PAGE, were combined, concentrated as described above, and the NaCl concentration diluted to 200 mM using 20 mM Hepes pH 7.5. Purified *Bs*Csp3 contained very little copper and typically had <1.3 equivalents of zinc when analyzed by AAS (n = 9). The mass of *Bs*Csp3 determined by FT-ICR-MS is 11838.1 Da, in very good agreement with the predicted value of 11838.7 Da for recombinant protein with Met1 and Ala108 respectively at its N- and C-termini.

Cloning the *copZ* gene

The *copZ* gene from *B. subtilis 168* was amplified from genomic DNA using the primers 5'-GCG**CATATG**GAACAAAAAACATTGC-3' (forward, *Nde*I restriction site in bold) and 5'-GCG**CCATGG**TCACTTGGCTACGTCATAGC-3' (reverse, *Nco*I restriction site in bold, stop codon underlined)[2]. Fragments were cloned into pGEM-T, verified by sequencing, and sub-cloned into the *Nde*I and *Nco*I sites to give pET29a-*Bs*CopZ.

Expression and purification of *Bs*CopZ

*Bs*CopZ was over-expressed and purified as described previously[2] with some modifications. *E. coli* BL21 (DE3) transformed with pET29a-*Bs*CopZ was grown in LB media at 37 ºC (100 µg/ml kanamycin) until an OD_{600} of ~0.6 when cells were induced with 1 mM IPTG, harvested after 5 h and stored at -30 ºC. Pellets were resuspended in 20 mM Tris pH 7.5, sonicated, and centrifuged at 40000 g for 25 min. The supernatant was diluted 5-fold in 20 mM Tris pH 7.5, loaded onto two connected HiTrap Q FF anion-exchange columns (5 ml) equilibrated in the same buffer, and proteins eluted with a linear NaCl gradient (0 to 400 mM, total volume ~240 ml). The *Bs*CopZ-containing fractions identified by SDS-PAGE were combined, diluted ~20-fold in 20 mM Mes pH 6.0 and loaded onto a HiTrap Q HP column (5 ml). Proteins were eluted using a linear NaCl gradient (0 to 1 M, ~180 ml) in 20 mM Mes pH 6.0. The purest fractions, identified by SDS-PAGE, were combined and thoroughly exchanged by ultrafiltration using an Amicon stirred cell with a 5 kDa molecular weight cut-off membrane into 20 mM Hepes pH 7.5 plus 200 mM NaCl. Concentrated *Bs*CopZ (~500 µM) was incubated with 10 to 30 mM ethylenediaminetetraacetic acid (EDTA), and further purified on a Superdex 75 10/300 GL column, equilibrated in 20 mM Hepes

pH 7.5 plus 200 mM NaCl. *Bs*CopZ fractions were combined, concentrated as described above, and stored at -30 °C. Purified *Bs*CopZ contained no copper or zinc, and has a mass of 7337.6 Da determined by FT-ICR-MS (the sample was treated with tris(2-carboxyethyl)phosphine prior to mass analysis as the two Cys residues were mainly oxidised in the purified protein), consistent with the calculated mass of 7338.1 Da (Met1 and Lys69 at the N- and C-termini respectively).

Isolation, purification and quantification of methanobactin. Full length apo-methanobactin (mbtin) from *M. trichosporium* OB3b was isolated, purified, and quantified as described previously[3].

Atomic absorption spectroscopy. AAS was carried out on an M Series spectrometer (Thermo Electron Corp.) typically with ten standards containing up to 1.8 ppm copper and 1.0 ppm zinc in 2% HNO_3 using the standard calibration method.

Protein quantification. Apo-Csp3 concentrations were determined using DTNB under anaerobic conditions as described previously[1]. For *Mt*Csp3 (final concentration ~0.15 to 2.0 μM) the reaction mixture typically contained ~480 μM DTNB in 20 mM Hepes pH 7.5 plus 200 mM NaCl, 1 mM EDTA and >7 M urea. At this urea concentration apo-*Mt*Csp3 rapidly unfolds, particularly at protein concentrations <6.5 μM (see below and Supplementary Fig. S2). Apo-*Bs*Csp3 unfolds more rapidly in guanidine hydrochloride than in urea (see below and Supplementary Fig. S3), and therefore thiol quantification was performed by the addition of apo-protein (final concentration ~0.2 to 2.9 μM) to DTNB (~450 to 480 μM) in 20 mM Hepes pH 7.5 plus 200 mM NaCl, 1 mM EDTA and ~6.0 M guanidine hydrochloride. For both proteins the absorbance at 412 nm reached a plateau after approximately 5 to 10 min (typically monitored for 20 min) when it was assumed that all 18 and 19 Cys residues respectively of unfolded apo-*Mt*Csp3 and apo-*Bs*Csp3 had reacted with DTNB (in the absence of denaturant the Cys residues are much less accessible and the reaction of apo-*Mt*Csp3 and apo-*Bs*Csp3 with DTNB after 20 min is ~7% and ~12% respectively of that with urea and guanidine hydrochloride present). An extinction coefficient of 14150 $M^{-1}cm^{-1}$ was used for the DTNB reaction in urea[1,4], whilst a value of 13000 M^{-1} cm^{-1} was used in the presence of guanidine hydrochloride[4]. The latter was verified using L-glutathione in 20 mM Hepes pH 7.5 plus 200 mM pH 7.5 with 1 mM EDTA and 6.5 M guanidine hydrochloride (n = 7). Apo-Csp3s (~4 to 20 μM) incubated overnight in an anaerobic chamber (Belle Technology, $[O_2]$ <<2 ppm) with DTT (~0.5 to 6.5 mM) in 20 mM Hepes pH 7.5 plus 200 mM NaCl and subsequently desalted on a PD10 column (GE Healthcare), sometimes twice, particularly at high DTT concentrations, were also quantified

using DTNB. Some samples that had been treated with DTT were re-quantified at various times after exposure to air at room temperature (*Mt*Csp3 samples left in air for up to 8 days (n = 3), and up to 21 days for *Bs*Csp3 (n = 3)). Apo-Csp3 concentrations were also determined with the Bradford assay (Coomassie Plus protein assay kit, Thermo Scientific) using BSA standards (0 to 1000 μg/ml), allowing a Bradford:DTNB concentration ratio to be determined. Treatment with DTT and exposure to air had no effect on the Bradford:DTNB ratio for both apo-*Mt*Csp3 and apo-*Bs*Csp3. DTT treatment was therefore excluded, particularly as contamination of apo-Csp3 with trace amounts of DTT would influence quantification using DTNB; the preferred approach due to its precision and sensitivity.

*Bs*CopZ has only two Cys residues in its $C^{13}XXC^{16}$ motif and the purified protein was largely oxidised. To fully reduce apo-*Bs*CopZ, the protein (35 to 350 μM) in 20 mM Hepes pH 7.5 plus 200 mM NaCl was incubated with DTT (4 to 20 mM) overnight in the anaerobic chamber and desalted twice on a PD10 column. The reduced protein was subsequently quantified using the DTNB assay as described above but in the absence of denaturant.

Investigating Cu(I) binding and the preparation of Cu(I) samples. 50 to 100 mM stock solutions of $[Cu(CH_3CN)_4]PF_6$ (Sigma, 97%) in 100% acetonitrile were typically diluted to ~1 to 13 mM (on one occasion to 51.9 mM to prepare a *Mt*Csp3 sample loaded with 17.4 equivalents of Cu(I) for unfolding studies followed by far-UV circular dichroism (CD) spectroscopy, *vide infra*) in 20 mM Hepes pH 7.5 plus 200 mM NaCl, all performed in the anaerobic chamber[5,6]. Copper and Cu(I) concentrations were determined by AAS and using the chromophoric high affinity Cu(I) ligand bathocuproine disulfonate (BCS) respectively[6-8]. For titrations, Cu(I) from the buffered solution was added to apo-Csp3 (~2 to 19 μM) in 20 mM Hepes pH 7.5 plus 200 mM NaCl under anaerobic conditions as described previously[1,5]. UV-Vis spectra were acquired on a λ35 spectrophotometer (Perkin Elmer), whilst emission spectra were acquired on a Cary Eclipse fluorescence spectrophotometer (Varian) by exciting at wavelengths between 250 and 310 nm and following the maximum emission in the 400 to 700 nm range using excitation and emission slits of 10 and 20 nm respectively. The concentration of Cu(I) solutions were regularly checked during titrations, usually with BCS, and replaced when required.

To prepare Cu(I)-Csp3 samples, the appropriate amount of a buffered Cu(I) solution was mixed in the anaerobic chamber with apo-protein (~9 to 360 μM for *Mt*Csp3 and ~35 to 130 μM for *Bs*Csp3) quantified by DTNB[1,5,6]. The protein concentration of Cu(I)-Csp3 samples cannot be determined using the DTNB assay due to very slow unfolding in the presence of denaturants (see below and Supplementary Figs S2 and S3). Therefore, the Cu(I) equivalents quoted are usually

based on apo-protein concentrations determined by DTNB, taking into account dilution of the protein due to Cu(I) addition, and are for Csp3 monomers.

Determination of Cu(I)-binding stoichiometry of the Csp3s. To determine the number of tightly bound equivalents per monomer, Cu(I) was added to apo-Csp3 in the presence of bicinchoninic acid (BCA) in 20 mM Hepes pH 7.5 plus 200 mM NaCl[1,6]. For *Mt*Csp3 this involved incubating mixtures (~2.4 to 2.9 μM of apo-protein plus ~100 μM BCA) in the anaerobic chamber with various concentrations of Cu(I). Equilibration occurred in ~3 h, with little variation over longer times (up to 67 h). For *Bs*Csp3 equilibration was faster and the experiment was performed by either titrating Cu(I) anaerobically into a mixture of apo-protein (~2.3 to 3.2 μM) plus BCA (~90 to 130 μM) and waiting up to 45 min for equilibration or by incubating mixtures in the anaerobic chamber for 1 to 4 h (typically 2.5 h, checked for up to 43 h). This experiment was also performed using apo-Csp3 samples (~10 to 20 μM) incubated overnight with DTT (~1 to 5 mM) in 20 mM Hepes pH 7.5 plus 200 mM NaCl and desalted (twice when higher DTT concentrations were used) and no difference was observed. All samples were analyzed for the formation of $[Cu(BCA)_2]^{3-}$ by UV-Vis spectrophotometry (at 562 nm) under anaerobic conditions[6].

Estimation of the average Cu(I) affinities of the Csp3s. BCS was primarily used to determine the average Cu(I) affinity. This is because, as observed previously[1] for *Mt*Csp1, the calculated maximum occupancies were low in the presence of higher concentrations of BCA. This effect is more significant for *Mt*Csp3 than *Mt*Csp1 and the maximum occupancy did not exceed 11 equivalents per monomer at 1205 μM BCA in a single experiment with this ligand that was incubated for up to 86 h. Csp3 (~2.5 μM, 2.09 μM on one occasion for *Bs*Csp3) in 20 mM Hepes pH 7.5 plus 200 mM NaCl was mixed anaerobically with increasing Cu(I) concentrations in the presence of 100 to 121 μM BCS. Samples were incubated anaerobically for up to 124 and 138 h for *Mt*Csp3 and *Bs*Csp3 respectively (apo-*Mt*Csp3 and apo-*Bs*Csp3 remain fully folded when left at room temperature in the anaerobic chamber for up to 409 and 228 h respectively, and Cu(I)-*Mt*Csp3 was found to be equally stable, see Supplementary Fig. S9). Affinity data are reported for mixtures after incubation for 42-43 h for *Mt*Csp3 and 90-96 h for *Bs*Csp3. The concentration of $[Cu(BCS)_2]^{3-}$ was determined by UV-Vis spectrophotometry (at 483 nm) and the concentration of Cu(I) bound to Csp3 calculated as described previously[1]. The fractional Cu(I) occupancy per monomer was determined using the maximum calculated value observed in a particular experiment and plots against the free Cu(I) concentration ($[Cu(I)_{free}]$) were fit to the non-linear form of the Hill equation

(1) to obtain an estimate of the average dissociation constant of Csp3 for Cu(I) (K_{Cu}) and the Hill coefficient (n value)[1,9,10].

$$\text{Fractional Cu(I) occupancy } = \frac{[\text{Cu(I)}_{\text{free}}]^n}{K_{\text{Cu}}^n + [\text{Cu(I)}_{\text{free}}]^n} \qquad (1)$$

The maximum calculated Cu(I) occupancies for a monomer ranged from 15.1 to 16.9 for *Mt*Csp3 in the presence of BCS (42 to 43 h incubation), 10.9 for *Mt*Csp3 in the presence of BCA (after 86 h incubation) and 18.1 to 18.4 for the experiments with *Bs*Csp3 plus BCS (90 to 96 h incubation). To check these values, appropriate Cu(I)-Csp3 samples were separated under anaerobic conditions from $[Cu(BCS)_2]^{3-}$ and free BCS (and also $[Cu(BCA)_2]^{3-}$ and free BCA) using a PD10 column in 20 mM Hepes pH 7.5 plus 200 mM NaCl and analyzed for Cu(I) using BCS (~2.4 mM) in the presence of 6.2 M guanidine hydrochloride, and for protein with the Bradford assay corrected using the Bradford:DTNB ratio of the apo-protein sample used in the particular experiment. Examples of the results for the experiments with BCS are given in the main manuscript. For the BCA experiment with *Mt*Csp3, 16.0 equivalents of Cu(I) were determined per monomer (10.1 to 10.9 equivalents calculated) for *Mt*Csp3 samples to which 32.6 to 41.0 equivalents of Cu(I) were incubated in the presence of 1205 μM BCA for 86 h. The cause of the low calculated occupancies with BCA is currently not known[1].

Cu(I) removal from Csp3. The ability of *M. trichosporium* OB3b mbtin to remove Cu(I) from *Mt*Csp3 was performed in 20 mM Hepes pH 7.5 plus 200 mM NaCl as described previously[1]. *Mt*Csp3 (0.96 and 0.94 μM) loaded with 18.0 equivalents of Cu(I) was added to apo-mbtin (17.1 and 33.9 μM respectively) and monitored by UV-Vis spectrophotometry for up to 360 h. Apo-*Mt*Csp3 and Cu(I)-*Mt*Csp3 remain fully folded when left at room temperature in the anaerobic chamber for up to 409 h, Supplementary Fig. S9). Furthermore, the UV-Vis spectrum of an apo-mbtin sample (31.0 μM) incubated in the anaerobic chamber in the same buffer is almost unaltered over this time period. Controls were performed by adding Cu(I) (16.7 μM) to 16.8 and 33.8 μM apo-mbtin and these mixtures were analyzed over 4 h. All samples were incubated anaerobically out of light. Gel-filtration chromatography was used to monitor Cu(I) exchange from *Bs*Csp3 to *Bs*CopZ (see below).

Cu(I) removal by BCS (~2.4 mM) was investigated for Csp3s (~0.4 to 1.3 μM) to which ~16 to 19 equivalents of Cu(I) were added The reaction was monitored anaerobically by UV-Vis spectrophotometry at 483 nm in 20 mM Hepes pH 7.5 plus 200 mM NaCl for typically 2 h in the presence of guanidine hydrochloride and for up to ~80 h with denaturant absent. No difference in

Cu(I) removal was observed using Cu(I)-Csp3 samples prepared from apo-protein previously incubated overnight with DTT (2 to 5 mM) and desalted.

Far-UV circular dichroism spectroscopy. Far-UV CD spectra (180 to 250 nm) were recorded using a JASCO J-810 spectrometer as described previously[8,11]. Spectra of apo-*Mt*Csp3 (17.9 to 39.9 μM, 0.26 to 0.58 mg/ml) and protein (22.7 to 46.8 μM, 0.33 to 0.68 mg/ml) loaded with ~18 to 19 equivalents of Cu(I) were measured in 100 mM potassium phosphate pH 8.0. The stability of apo-*Mt*Csp3 (41.4 μM, 0.60 mg/ml) and protein (33.6 μM, 0.49 mg/ml) loaded with 16.9 Cu(I) equivalents was monitored for up to 409 h, with the final pH within 0.1 pH unit of that expected. Unfolding of apo-*Mt*Csp3 (27.5 μM, 0.40 mg/ml and 6.44 μM, 0.094 mg/ml) was investigated in the presence of 6.7 M urea for up to 1 h. Unfolding of *Mt*Csp3 (7.28 μM, 0.11 mg/ml and 29.1 μM, 0.42 mg/ml) loaded with 17.4 equivalents of Cu(I) in the presence of 6.2 M guanidine hydrochloride was monitored for up to 24 h.

Far-UV CD spectra of apo-*Bs*Csp3 (37.9 to 47.6 μM, 0.45 to 0.56 mg/ml) and protein (35.5 to 43.4 μM, 0.42 to 0.51 mg/ml) to which 19.3 to 19.7 equivalents of Cu(I) were added were acquired in 100 mM potassium phosphate pH 8. The stability of apo-*Bs*Csp3 (41.4 and 43.9 μM, 0.49 and 0.52 mg/ml) was monitored for up to 228 h and the final pH was within 0.1 pH unit of that expected. Unfolding of apo-*Bs*Csp3 (41.9 μM, 0.50 mg/ml) was monitored in the presence of 6.0 M guanidine hydrochloride in 20 mM Hepes pH 7.5 plus 200 mM NaCl for up to 60 min. Unfolding of *Bs*Csp3 (8.45 μM, 0.10 mg/ml) to which 18.8 equivalents of Cu(I) were added was monitored in the presence of 6.0 M guanidine for up to 42 h.

For both proteins stability and unfolding samples analyzed by far-UV CD were in 20 mM Hepes pH 7.5 plus 200 mM NaCl and were prepared and subsequently incubated in the anaerobic chamber. The α-helical content was determined using the mean residue ellipticity at 222 nm[12].

Analytical gel-filtration chromatography of the Csp3 proteins. Analytical gel-filtration chromatography of apo-Csp3 (~3 to 140 μM) and protein (~2 to 110 μM) to which ~16 to 21 equivalents of Cu(I) were added was performed on a Superdex 75 10/300 GL column. The elution buffer was 20 mM Hepes pH 7.5 plus either 200 (*Mt*Csp3) or 500 (*Bs*Csp3) mM NaCl (all samples loaded in 20 mM Hepes pH 7.5 plus 200 mM NaCl) and in some cases this was thoroughly degassed and purged with nitrogen throughout the experiment[1,5]. The column was calibrated at the two different NaCl concentrations used allowing apparent molecular weights to be calculated from elution volumes for apo- and Cu(I)-Csp3s. Experiments with apo-Csp3 were also performed on samples (~4 to 100 μM) incubated overnight in the anaerobic chamber with DTT (~40 to 120 μM

protein and ~4 to 9 mM DTT), with chromatography performed in the same buffers as above plus 1 mM DTT. Injection volumes were 100 μl, the flow rate was 0.8 ml/min and absorbance was monitored at 240 nm.

A mixture of *Bs*Csp3 (1.50 to 3.40 μM, n = 3) loaded with 15.1 to 17.7 equivalents of Cu(I) was mixed with apo-*Bs*CopZ (48.0 to 100 μM) in 20 mM Hepes pH 7.5 plus 200 mM NaCl. At various time points 500 μl of this mixture was injected onto the Superdex 75 10/300 GL column equilibrated with thoroughly degassed 20 mM Hepes pH 7.5 plus 500 mM NaCl that was bubbled with nitrogen. Elution was performed in degassed and nitrogen bubbled 20 mM Hepes pH 7.5 plus 500 mM NaCl, monitored at 240 nm and the copper content of the eluted *Bs*Csp3 peak quantified by AAS. Control samples of apo- and Cu(I)-*Bs*Csp3 were also tested. This experiment was performed three times and data from an experiment at 3.40 μM *Bs*Csp3 plus 15.1 equivalents of Cu(I) in the presence of 100 μM apo-*Bs*CopZ is shown in Fig. 5b, giving ~45 and 33% Cu(I) loss from *Bs*Csp3 after 64 h using the absorbance at 240 nm and AAS data respectively. For an experiment at 1.50 μM *Bs*Csp3 plus 17.7 equivalents of Cu(I) in the presence of 48.3 μM apo-*Bs*CopZ copper removal after 41 h was found to be ~38 and 48% determined from the absorbance at 240 nm and by AAS respectively. When the experiment was performed at 3.00 μM *Bs*Csp3 plus 17.0 equivalents of Cu(I) in the presence of 100 μM apo-*Bs*CopZ copper removal after 41 h was determined to be ~38 and 42% from the absorbance at 240 nm and by AAS respectively.

Crystallisation, data collection, structure solution and refinement. Apo-*Mt*Csp3 (~20 mg/ml, Bradford assay) in 20 mM Hepes pH 7.5 and apo-*Bs*Csp3 (~20 mg/ml, DTNB quantification) in the same buffer plus 200 mM NaCl were crystallised aerobically at 20 ºC using the sitting drop method of vapor diffusion. Apo-*Mt*Csp3 (100 nl) was mixed with 100 nl of 40% pentaerythritol propoxylate (5/4 PO/OH), 100 mM Hepes pH 7 and 200 mM sodium thiocyanate (80 μl well solution) and resulting crystals were frozen in 20% PEG 400. Crystals of apo-*Bs*Csp3 were obtained from a 1:1 mixture (200 nl) with 100 mM citrate pH 4 plus 800 mM ammonium sulfate (80 μl well solution), and were frozen in saturated ammonium sulfate. Apo-*Mt*Csp3 (70 μM, DTNB quantified) plus 18.0 equivalents of Cu(I) in 20 mM Hepes pH 7.5 plus 200 mM NaCl, was concentrated (~17 mg/ml, Bradford assay) and crystallised anaerobically using the hanging drop method of vapor diffusion. Diffraction-quality crystals were obtained by mixing 1 μl protein with 1 μl 200 mM $MgCl_2$, 100 mM Hepes pH 7.5, and 36% PEG 400 (500 μl well volume) and were directly frozen.

All crystallographic data were collected at Diamond Light Source Ltd, UK, beamline I02. Apo-*Mt*Csp3 data were integrated with MOSFLM[13]. Apo-*Bs*Csp3 and Cu(I)-*Mt*Csp3 data were integrated with XDS[14]. All data were scaled with Aimless[15] and space group determination was

confirmed with Pointless[16]. The structure of apo-*Mt*Csp3 was solved by molecular replacement using Molrep implemented via the CCP4 suite[17] with PDB file 3KAW (apo-Csp3 from *Pseudomonas aeruginosa*, deposited in the PDB but functionally uncharacterized) as the search model. The structures of Cu(I)-*Mt*Csp3 and apo-*Bs*Csp3 were solved by molecular replacement using Molrep with apo-*Mt*Csp3 as the search model. The initial solution for apo-*Bs*Csp3 was improved using the automated model building program ARP/wARP[18]. All models underwent cycles of model building in Coot[19] and refinement in REFMAC5[20]. Five percent of observations were used to monitor refinement. All models were validated using MolProbity[21] and data collection statistics and refinement details are reported in Supplementary Table S1.

Studies using the *copA* deletion strain of *E. coli*. *E. coli* BW25113 and the strain in which the CopA-encoding gene has been inactivated through allelic replacement (herein Δ*copA*)[22] were obtained from the Coli Genetics Stock Center. The published phenotype for Δ*copA* relative the wild type (WT) strain[22] was confirmed using overnight cultures grown in LB (plus 50 μg/ml kanamycin for Δ*copA*) and diluted 100-fold in LB and LB plus copper nitrate (0.5 to 2.0 mM) at 37 ºC (agitation 250 rpm) and measuring the OD at 600 nm at regular intervals up to 6 h (see Fig. 6a). WT and Δ*copA* were tested by PCR with the following primers; 5'-CCGATTTTTTAATCTTTACGGAC-3' and 5'-GCGTCTTATCAGGCCTACAAACCTG-3', designed to hybridise 100 bp upstream and downstream of the *copA* gene giving PCR fragments of 2754 and 1571 bp respectively. *Bs*Csp3 was sub-cloned from pET29a into the *Xba*I and *Hind*III sites of pBAD33 giving pBAD33_*Bs*Csp3. Δ*copA* transformed with pBAD33_*Bs*Csp3 was grown overnight at 37 ºC (agitation 250 rpm) in the presence of kanamycin (50 μg/ml) and chloramphenicol (30 μg/ml) and then diluted 100-fold into LB and LB plus copper nitrate (0.5 to 2.0 mM) without antibiotics but in the presence of 0.2% arabinose. The OD at 600 nm was measured relative to Δ*copA* and Δ*copA* containing pBAD33 grown overnight with kanamycin (50 μg/ml) and kanamycin (50 μg/ml) plus chloramphenicol (30 μg/ml) respectively, and diluted 100-fold in LB plus copper. Cell cultures (~35 ml) were collected by centrifugation (5000 g at 4 ºC for 10 min) after 12 h growth and frozen at -30 ºC. Thawed cells were washed twice with 20 ml of 20 mM Tris plus 10 mM EDTA and 100 mM NaCl and then with 20 ml of 20 mM Tris plus 100 mM NaCl. The cells were digested in 200 μl of 65% nitric acid for up to three days, centrifuged at 12000 g for 10 min at room temperature, diluted in MilliQ water to give a final nitric acid concentration of 2% and analyzed for copper by AAS.

Studies using the *csp3* deletion strain of *B. subtilis*. The *B. subtilis* 168 strain with the *Bs*Csp3-encoding gene (*yhjQ*) inactivated through allelic replacement (herein Δ*csp3*) was obtained from the Bacillus Genetic Stock Centre library. Cultures were grown (agitation 250 rpm) in LB at 37 ºC overnight, and diluted (~100-fold) in LB and LB plus copper nitrate (0.1 to 4 mM) and grown for up to 32 h at 37 ºC with the OD at 600 nm measured at regular intervals. All WT and Δ*csp3* cultures used for the data shown in Supplementary Fig. S11 were tested by PCR with the primers used to clone *Bs*Csp3 and also using the following primers; 5'-CATTCATGACAGTGCGACG-3' and 5'-CACAAGAGGACTGGACGC-3', designed to hybridise ~ 300 bp upstream and downstream of the Csp3 gene giving a PCR fragment of 967 bp for WT and longer for the Δ*csp3* strain (~1.5 kbp).

Supplementary References

1. Vita, N. *et al*. A four-helix bundle stores copper for methane oxidation. *Nature*, **525**, 140–143 (2015).
2. Kihlken, M. A., Leech, A. P. & Le Brun, N. E. Copper-mediated dimerization of CopZ, a predicted copper chaperone from *Bacillus subtilis*. *Biochem. J.* **368**, 729–739 (2002).
3. El Ghazouani, A. *et al*. Copper-binding properties and structures of methanobactins from *Methylosinus trichosporium* OB3b. *Inorg. Chem.* **50**, 1378–1391 (2011).
4. Riener, C. K., Kada, G. H. & Gruber, J. Quick measurement of protein sulfhydryls with Ellman's reagent and with 4,4'-dithiodipyridine. *Anal. Bioanal. Chem.* **373**, 266–276 (2002).
5. Badarau, A., Firbank, S. J., McCarthy, A. A., Banfield, M. J. & Dennison, C. Visualizing the metal-binding versatility of copper trafficking sites. *Biochemistry* **49**, 7798–7810 (2010).
6. Allen, S., Badarau, A. & Dennison, C. Cu(I) affinities of the domain 1 and 3 sites in the human metallochaperone for Cu,Zn-superoxide dismutase. *Biochemistry* **51**, 1439–1448 (2012).
7. Badarau, A. & Dennison, C. Copper trafficking mechanism of CXXC-containing domains: insight from the pH-dependence of their Cu(I) affinities. *J. Am. Chem. Soc.* **133**, 2983–2988 (2011).
8. Badarau, A. & Dennison, C. Thermodynamics of copper and zinc distribution in the cyanobacterium *Synechocystis* PCC 6803. *Proc. Natl. Acad. Sci. U.S.A.* **108**, 13007–13012 (2011).
9. Xiao, Z., Loughlin, F., George, G. N., Howlett, G. J. & Wedd, A. G. C-terminal domain of the membrane copper transporter Ctr1 from *Saccharomyces cerevisiae* binds four Cu(I) ions as a cuprous-thiolate polynuclear cluster: sub-femtomolar Cu(I) affinity of three proteins involved in copper trafficking. *J. Am. Chem. Soc.* **126**, 3081–3090 (2004).

10. Banci, L. *et al.* Affinity gradients drive copper to cellular destinations. *Nature* **465**, 645–648 (2010).
11. Allen, S., Badarau, A. & Dennison, C. The influence of protein folding on the copper affinities of trafficking and target sites. *Dalton Trans.* **42**, 3233–3239 (2013).
12. Barrow, C. J., Yasuda, A., Kenny, P. T. M. & Zagorski, M. G. Solution conformations and aggregational properties of synthetic amyloid β-peptides of Alzheimer's Disease. Analysis of circular dichroism spectra. *J. Mol. Biol.* **225**, 1075–1093 (1992).
13. Leslie, A. G. W. & Powell, H. R. Evolving methods for macromolecular crystallography. 245, 41-51 ISBN 978-1-4020-6314-5 (2007).
14. Kabsch, W. XDS, *Acta Cryst*. **D66**, 125–132 (2010).
15. Evans P. R. & Murshudov G. N. How good are my data and what is the resolution? *Acta Cryst.* **D69**, 1204–1214 (2013).
16. Evans P. R. Scaling and assessment of data quality. *Acta Cryst*. **D62**, 72–82 (2006).
17. Winn, M. D. *et al*. Overview of the CCP4 suite and current developments. *Acta. Cryst.* **D67**, 235–242 (2011).
18. Langer, G., Cohen, S. X., Lamzin, V. S., & Perrakis A. Automated macromolecular model building for X-ray crystallography using ARP/wARP version 7. *Nat. Protoc.* **3**, 1171–1179 (2008).
19. Emsley, P., Lohkamp, B., Scott, W. G., & Cowtan, K. Features and development of Coot. *Acta Cryst.* **D66**, 486–501 (2010).
20. Vagin, A. A. *et al*. REFMAC5 dictionary: organisation of prior chemical knowledge and guidelines for its use. *Acta Cryst*. **D60**, 2284–2295 (2004).
21. Chen, V. B. *et al*. MolProbity: all-atom structure validation for macromolecular crystallography. *Acta Cryst*. **D66** (Pt 1), 12–21 (2010).
22. Rensing, C., Fan, B., Sharma, R., Mitra, B. & Rosen, B. P. CopA: an *Escherichia coli* Cu(I)-translocating P-type ATPase. *Proc. Natl. Acad. Sci. U. S. A.* **97**, 652–656 (2000).
23. Notredame, C., Higgins, D. G. & Heringa, J. T-Coffee: a novel method for fast and accurate multiple sequence alignment. *J. Mol. Biol.* **302**, 205–217 (2000).
24. Kau, L. S., Spira-Solomon, D. J., Penner-Hahn, J. E., Hodgson, K. O. & Solomon, E. I. X-ray absorption edge determination of the oxidation state and coordination number of copper: applications to the type 3 site in *Rhus vernicifera* laccase and its reaction with oxygen. *J. Am. Chem. Soc.* **109**, 6433–6442 (1987).

```
Brevibacterium      MTH------------ITSMIET-------HPND-TTGLDVQ-K-LA----------DCIAACF
Corynebacterium     MEHDMT-------HHVRTMLDT-------HPKD-LGQIDKD-K-LA----------ECIEACF
Micrococcus         MTHH-----------VSAMLET-------HPKD-LGGIDRR-T-LA----------ECIEACL
Mycobacterium       MAVAM--------------------------------V-EK-T-NR----------ACLDACV
Rhodococcus         M------------------LES-------YPKN-VGEIDTG-V-LA----------ACIEACI
Streptomyces        MPTT-----------VNDLLRT-------YPAD-LGGVDRE-A-MA----------RCIEECL
Bacillus cereus     MQ--------------------------------------N-M-YQ----------ACIEECL
Bacillus subtilis   ME--------------------------------------Q-Y-SE----------ACIEACI
Lysinibacillus      MN--------------------------------------M-S-LE----------ECIKACL
Marinococcus        MSVN------------------------------------A-A-QK----------ECLEACI
Staphylococcus      MS--------------------------------------YGK-NR----------LLIQTLH
Streptococcus       MTHH-----------VSAMLDT-------YPKN-VGNIDRQ-K-LA----------ECIQACF
Anabaena            MSIQEL--------------------------T-LSQV-NQ-Q-MQ----------QCIQNCL
Calothrix           MMML-----------KTE------------------SM-TT-E-IQ----------TCLDAIT
Synechocystis       MTHHLA----------------------------LYQV-NP-E-MK----------QCIQNCL
Rudanella           MI-------------------------------------AQ-H-FQ----------DCIDACQ
Sphingobacterium    MSH-------------------------------------Q-Q-FQ----------KCIDECY
Nitrospira          MT--------------------------------------E-Q-YK----------MCISACQ
Gimesia             MS----------------------------------------L-KQ----------SCIDACL
Phycisphaera        MK------------------------------------------NA----------DCIQTCL
Bradyrhizobium      MESSMQ---------AQAMIST-------HPQV-RGNT-ND-A-LI----------RCIEECY
Methylosinus        MHVE-------------AMISK-------HPQA-RGQT-DR-S-LV----------QCVEMCF
Rhizobium           MTMH-----------H---------------------M-ST-E-MK----------ACIDNCL
Rhodospirillum      MSLR-------------EMIAD-------HPDV-RGDL-NP-A-LA----------ACIEETL
Cupriavidus         MIRPTV----------------------------Q--ENAA-R-YA----------DCIAACN
Nitrosomonas        MFLYTE--------------------------------TDQ-N-LQ----------ACIDACN
Ralstonia           MIRPTV----------------------------Q--ENFS-R-YA----------DCIAACN
Thiobacillus        MH--------------------------------------T-E-FA----------SCIKACD
Azotobacter         M-N-------------------------------------A-SMYE----------SCIQACS
Klebsiella          M-P-------------------------------------V-N-YA----------ECIEVCY
Legionella          MTH-------------------------------------Q-Q-YD----------MCIKACQ
Methylomicrobium    MYTPQT-------E----------------PQK-TSTAAFQ-S-MQ----------PCIDNCN
Pseudomonas         MTRAI-----------NDPGNE-------DPGS-LLETDAD-A-LLGGAAAQAPEERCRLAAQ
Salmonella          M-Q-------------------------------------Q-E-HR----------ECIEQCY
Bdellovibrio        MIPNP-----------------------------S--Q-DS-D-TE----------KAIKSNF
Desulfovibrio       MFRA------------RDMLEH-------H-KA-AGRMDRE-S-LV----------RCIEACY
Myxococcus          MAPAE-----------------------------VMKT-DS-D-LH----------QCIEDCL
Deinococcus         MTNPMT-------QPLQGMLET-------HPQAGQGNLDQQ-A-LL----------ECLAACF
Leptospira          MNRKELLQKAGMAVAVSGILSTLSAEDHDHSTA-MPTAGKS-K-YA----------KAMMAAI
Chthoniobacter      MDP-------------------------------------E-K-YQ----------ACIEACH
Opitutus            MPH-------------------------------------Q-K-YQ----------DCIEACH
Sphaerobacter       MAHHTS----------------------------RHGM-DD-Q-MA----------DAIAVVN
Ktedonobacter       MHEP-----------I---------------------I-ES-H-LQ----------QYTQACN
consensus           *


Brevibacterium      ECAQTCTA-CA-DACLAE---D-MVAELRNCIRLNLDCADLCAATGSILSRRTGQNLATVKAA
Corynebacterium     ECAQTCTA-CA-DACLGE---D-MVAELTTCIRLNLDCADICDVTGRVLSRQTGWDVNLIRSV
Micrococcus         ACAQTCTA-CA-DACLSE---D-VVAEMRRCIRLNLDCADVCGATAAVLTRQTGEDASTVRAL
Mycobacterium       ECQQACET-CN-YNCCAQ---D-GQ--MAECARLCLDCAAICAACVTLLSRGS----RWAAQL
Rhodococcus         DCAQTCTA-CA-DACLAE---D-TVAELTACIRTDLDCADLCAATARILSRQTGNNAAVTKAV
Streptomyces        RCAQACTA-CA-DACLSE---P-TVADLTKCIRTDMDCADVCTATAAVLSRHTGYDANVTRAV
Bacillus cereus     KCMEICNS-CY-SACLQE---S-DVKMMVECIRLDRECADICALAAKSMSSNS----PFAKDI
Bacillus subtilis   DCMKACNH-CF-TKCLEE---S-VQHHLSGCIRLDRECADICALAVKAMQTDS----PFMKEI
Lysinibacillus      ECMEACNG-CY-DACLKE---E-DVKMLAECIRSLRECADICAFSAKSMQSDS----PYVKQI
Marinococcus        ECMKQCNQ-CF-NDCLQE---D-DVKMMAECIRLDRECADICALTAKAIQSNS----PLMKET
Staphylococcus      ECVEACNY-CF-QACLKE---E-NVKMMAECIRLDRECADMCTFLERELTIDS----PFAYDL
Streptococcus       ECAQTCTA-CA-DACLAE---D-MVADLRQCIRLNLDCADVCAATGRMLSRQTGNNVETTRAL
Anabaena            DCHSICLN-TV-TYCLQK---G-GHHAKPAHIRLMLDCAEICNTSANFMLRAS----DLHSRT
Calothrix           ECQKACLE-SM-AHCMSK---G-GKYIDVSVMSTMRDCAEMCMMCANMMRGSS----EFVQRT
Synechocystis       DCHSICLN-TV-TYCLQQ---G-GMHSELPHIRLMLDCAEICQTSANFMLRGS----ETCIRT
Rudanella           ACALACDQ-CA-DACLGE---E-HVHHMVECIRLDRDCAKICQMAVSFMASNS----AHAADV
Sphingobacterium    ACAVACNH-CA-ASCLQE---D-NVKMMARCIQLDLECAAICRAAAELMSLGS----QYSRHL
```

```
Nitrospira          SCAGICNT-CS-DDMIGMEPHG-DRELMARCIRLCRECADICSLSVNWMNRLS----PLAESL
Gimesia             KCATDCEF-CL-DAMIGK---E-S---DNDCPHCCRECVDICLYTAQALARNS----KYSSQI
Phycisphaera        ECAADCDH-CL-AKMAGM-------ESSNDCPACCIDCAAACRACATLLASEG----PFSREA
Bradyrhizobium      SCAQTCTS-CA-DACLGE---D-KVQSLTQCIRLNMDCADICNITGRIATRRTGSDEEMIRRM
Methylosinus        DCAQTCAA-CA-DACLGE---D-KVADLRHCIRLNLDCAEICVAAGSIASRAAGTEESILRTM
Rhizobium           ACYRECLS-MAMGHCLEL---G-GEHTKPQHFKLMMACAEICRTSAHFMLIGS----EHHKHV
Rhodospirillum      ACAQVCTA-CA-DACLAE---G-MVAELRQCIRLNLDCADVCAAAGAVATRRTGGNVPVIRAL
Cupriavidus         AAAAAALK-CA-AACLEE---Q-DVRKMARCIALDMDCAGIAQLAASYMLRNS----EFAPLV
Nitrosomonas        HCYRTCLRMAM-NHCLEA---G-GKHVEADHLRLMMNCAEICQTSLNFMLSGS----RFSPKV
Ralstonia           AAAAACLK-CA-AACLEE---P-DTRKMTRCIALDMDCAGIANLAASYMLRNS----EFAPLV
Thiobacillus        ECAAACDH-CA-TACLQE---A-DPKPMARCIALDIDCAAICRLASGYMARGS----EFARRM
Azotobacter         DCAWSCET-CA-ASCLRE---D-DVQAMARCISLDMDCADFCRMAATLMARGS----DHAPAF
Klebsiella          KCATACDY-CA-ASCLKE---E-QVDMMRECIRLDMQCANICRLAAQFMTLDS----EFAKSL
Legionella          ACLLECEH-CA-NACLHE---E-DCNDLARCISLDRDCAAICALAIEMMARNS----PFAKEI
Methylomicrobium    RCAQTCLQTAM-NQCLEM---G-GRHVEPEHFRLMICCAEICRLSANFMLSSS----PFHTRT
Pseudomonas         ACIRACERY-LA-LCT---------ESSREQRQHAGDCADLCRLAALLLERRS----PWAPAA
Salmonella          ECAAACDI-CA-SSCLRE---D-NVEMMKHCIQLDMQCAAICRLAAQFMALES----EYSQKL
Bdellovibrio        TCSRVCLET-L-QYCLNQ----KSIKFSGQHLAVMQFCADACLLSARMMMANH----SVHHQS
Desulfovibrio       ECAQTCAM-CA-DACLAE---D-EVKRLARCIRLDLDCMDICVATGNALSRQVGVEPAIQRAQ
Myxococcus          ACHRVCVE-TL-TYCLSK---G-GRHAEAGHLRLLMDCAEICQTSANFMLRGS----ELHSRT
Deinococcus         ECAQVCTS-CA-DACLGE---Q-NLDMLRRCIRLNLDCADVCDATGRVLTRCTQPDMNVVRTQ
Leptospira          HCQLSAEV-CLS-HCITEL--GKGDKAMAACAASTREVISLCDSFVKLASQNS----SFTKKL
Chthoniobacter      ACVTACEN-CA-ASCLEE---D-DVEMMVGCIELDRSCADICALAAREMARDS----DFAVRV
Opitutus            KCAVECEH-CA-TACLHE---D-DIKMMVRCIELDRSCADICALAEREMARGS----EFAERV
Sphaerobacter       ECHDTCLT-TI-SYCLEH---G-GEHAKAEHIRLMLDCVDICRTAAAFMLRDS----ALEGRV
Ktedonobacter       YCHAVCEQ-TL-HYCLHK---G-GRLLQGDCVQALIDCSQICQLTQDLTLRQS----PLQERA
consensus            .   .                                   .

Brevibacterium      LEACRTACAECAAECEKHADMHEHCRVCAEACRRCEQACADLLAAI-----G----------
Corynebacterium     LETCRAACQACGEECARHADMHEHCKVCAEACRRCEQACAELLATL-----A----------
Micrococcus         LEACRNACRACGDECASHAEMHEHCAVCAESCRRCEQACADLLASL-----G----------
Mycobacterium       CQLCAQVCDACAAECDKYDR--DYCRECAQACRRCAEQCRSMA-G-----------------
Rhodococcus         LEACATACRVCGDECDRHSSHHEHCRICAEACRRCEQACRDLLAAL-----D----------
Streptomyces        LQACATVCAACGDECARHAGMHEHCRVCAEACRSCEQACQELLAGL-----G----------
Bacillus cereus     CQLCAKICEACGNECKKHE--HQHCKECADACFRCAEACKQMV---S---------------
Bacillus subtilis   CALCADICEACGTECGKHD--HDHCQACAKACFTCAEQCRSMA---A---------------
Lysinibacillus      CQACAEICQACGDVCKKHEH-HEHCKQCAESCYKCAEVCRKMA---V---------------
Marinococcus        AQLCAEACKACGDECAKHD--HQHCKDCADACYRCEKACRELAA--S---------------
Staphylococcus      AEICSKICEACGNECQKHE--HDHCQECAKSCFKCAKACKDI----A---------------
Streptococcus       LEACRAACKTCGDECESHAQMHEHCKVCAEACRRCEQACADLLVTL-----G----------
Anabaena            CGVCAELCQRCADECDAYGD-DAQMKACAQMCRRCADSCRQMAMAT----------------
Calothrix           CMLCYQTCEKCVAACESWSD-DKKIMECIVACRKCAQYCKVISTVSH----H----------
Synechocystis       CAICAEICLKCAEDCDRMGD-DEQMKACAQMCRTCAETCRQMSMSMA---------------
Rudanella           CKLCAHICEACAAECGKHD--EDHCRACAEACRRCAEACRQMALSAHN--LN----------
Sphingobacterium    CKLCAEICRACGDECAKHDM--QHCKECADACYKCAEACEQMATAV----------------
Nitrospira          CRLCADVCDACAEACEQHAPHHELCGPCAEECRRCASICREMAGAAA---------------
Gimesia             SQLCADICTWCAEQCEAHE--HYHCQACAASCRKCAAECKEIVTV-----------------
Phycisphaera        CELCAKACERCAENCEQHD--HEHCKTCADSSRRCAEACRAMAA------------------
Bradyrhizobium      LEACTAVCRLCAEECERHAEMHEHCRICADACRRCMSACEEARPSLT----H----------
Methylosinus        LQTCAEMCRMCEEECRRHAGNHEHCRICADVCKECETACRSATGLT-----H----------
Rhizobium           CRECAEICGQCAEDCERIGD----MQSCVDACRRCADSCRKMAA------------------
Rhodospirillum      LDACALACRRCGEECARHAGAHAHCRLCADSCRRCEAACREALESLP----A----------
Cupriavidus         CEDCAEVCKWCKEECERHDA--EHCQECARACAVCMEQCLKMT----------------A--
Nitrosomonas        CGVCAEICDACAKSCEQLDG----MEECVQTCRQCAEHCRKMA-------------------
Ralstonia           CEDCAEVCKWCKEECERYD--HWHCQECAKACAACMEMCLKMT----------------A--
Thiobacillus        CAICAEVCEACGAECAKHQ--HDHCQECAQACRRCAEECRRMA----------------A--
Azotobacter         CRQCAQVCRACAEECARHEA--GHCRRCAQACRACAEECERMA--A----------------
Klebsiella          CQVCAEICQKCGEECGKHEA--EHCQKCSEACLRCAETCRSM---V----------------
Legionella          CALCAKICRACGDECSKHQHM-EHCQRCAKACYQCAEACEKMA-------------------
Methylomicrobium    CEVCAEICEACAKDCASIGD----MDECVSICRECAESCKQMASMAT---------------
Pseudomonas         CELAARYALACAERCDGDEP---LERECAGACRRFVEACRPLLPA-----------------
Salmonella          CRLCADICKACAEECARHD--HDHCQNCARACSQCADACLKMA--A----------------
```

```
Bdellovibrio     CELSFELCTACADECERHQD-DPVIARCAEECRRCAEICKSMVGMSVDI-RGPEGKREKTSTR
Desulfovibrio    VEACMEACRVCADECDKHAKMHEHCRICSEMCDTCKNACKVLLGNLEAK-AA----------
Myxococcus       CFACSEVCKRCAESCGRMGD-DVVMKACADMCTRCSDSCWKMGGGVMPQTPN-PEAAQRAADL
Deinococcus      LQACLAACEACGAECEQHAEHHQHCAICAESCRRCADACRNLLSGIS----A-----------
Leptospira       ANLCVEVCEACAKECDKHANHHAVCKECRDSCLACAKELKK---V-----------------
Chthoniobacter   CAICAEVCEACGTECGRHKM--DHCQHCAAACRRCAKLCLEMSQAHSSQ-P---------L--
Opitutus         CQVCAEICQACGDECAKHKM--DHCQRCAEACHRCAEACREMAGATAGG-RS-MTDKLLGR--
Sphaerobacter    CAVCAEVCDACAADCERFTG-DERMQACAEVCRRCAESCRQMAASA----------------
Ktedonobacter    RDLCAEACHRCTQTCQQFQE-DAQMHACANMCQQCATACQ----------------------
consensus           .   .  *   *            *   .
```

Supplementary Figure S1. Multiple sequence alignment of Csp3 from a range of bacteria produced using T-coffee[23]. Asterisks indicate fully conserved sequence positions; the ':' and '.' symbols indicate strongly and weakly similar sequence positions respectively. Cys residues are highlighted in yellow, and the residues corresponding to Asn58 (green), His104, His108 and His110 (cyan) in *Mt*Csp3 are also highlighted. The organism labels are coloured according to phylum: Actinobacteria (light blue), Firmicutes (black), Cyanobacteria (dark green), Bacteroidetes (purple), Nitrospirae (yellow), Planctomycetes (dark gray), Proteobacteria (dark blue), Deinococcus-Thermus (light green), Spirochaetes (orange), Verrucomicrobia (light gray), Chloroflexi (red). The sequences used are: Brevibacterium - *Brevibacterium senegalense*, accession WP_019157842; Corynebacterium - *Corynebacterium halotolerans*, accession WP_03499189; Micrococcus - *Micrococcus luteus*, accession WP_017489202; Mycobacterium - *Mycobacterium gordonae*, accession WP_055580843; Rhodococcus - *Rhodococcus rhodochrous*, accession WP_026061417; Streptomyces - *Streptomyces coelicolor*, accession NP_627493; Bacillus cereus - *Bacillus cereus*, accession WP_001180615; Bacillus subtilis (*Bs*Csp3, underlined) - *Bacillus subtilis*, accession WP_009966950; Lysinibacillus - *Lysinibacillus manganicus*, accession WP_036188714; Marinococcus - *Marinococcus halotolerans*, WP_040880959; Staphylococcus - *Staphylococcus xylosus*, accession WP_017722281; Streptococcus - *Streptococcus pneumoniae*, accession CVN95604; Anabaena - *Anabaena* sp. PCC7120, accession BAB77277; Calothrix - *Calothrix* sp. PCC7103, accession WP_019495439; Synechocystis - *Synechocystis* sp. PCC7509, accession WP_009630903; Rudanella - *Rudanella lutea*, accession WP_044129390; Sphingobacterium - *Sphingobacterium spiritivorum*, accession WP_002993225; Nitrospira - *Nitrospira moscoviensis*, accession WP_053382062; Gimesia - *Gimesia maris*, accession WP_002649334; Phycisphaera - *Phycisphaera mikurensis*, accession WP_014437624; Bradyrhizobium - *Bradyrhizobium japonicum*, accession WP_028137163; Methylosinus - *Methylosinus trichosporium* OB3b (*Mt*Csp3, underlined), accession WP_003608458; Rhizobium - *Rhizobium leguminosarum*, accession WP_062945174; Rhodospirillum - *Rhodospirillum centenum*, accession WP_012568141; Cupriavidus - *Cupriavidus taiwanensis*, accession WP_018006016; Nitrosomonas - *Nitrosomonas multiformis*, accession ABB75042 (PDB ID = 3LMF); Ralstonia - *Ralstonia metallidurans*, accession YP_587881; Thiobacillus - *Thiobacillus denitrificans*, accession WP_011313254; Azotobacter - *Azotobacter*

vinelandii, accession WP_061290871; Klebsiella - *Klebsiella pneumoniae*, accession KMB50080; Legionella - *Legionella pneumophila*, accession WP_015445062; Methylomicrobium - *Methylomicrobium album*, accession EIC28528; Pseudomonas - *Pseudomonas aeruginosa*, accession WP_003113280 (PDB ID = 3KAW); Salmonella - *Salmonella typhimurium*, accession NP_460228; Bdellovibrio - *Bdellovibrio bacteriovorus*, accession WP_025307894; Desulfovibrio - *Desulfovibrio africanus*, accession WP_005987168; Myxococcus - *Myxococcus fulvus*, accession WP_013935850; Deinococcus - *Deinococcus peraridilitoris*, accession AFZ67215; Leptospira - *Leptospira biflexa*, accession WP_012476553; Chthoniobacter - *Chthoniobacter flavus*, accession WP_006980546; Opitutus - *Opitutus terrae*, accession WP_012377177; Sphaerobacter - *Sphaerobacter thermophilus*, accession WP_012873578; Ktedonobacter - *Ktedonobacter racemifer*, accession WP_007919176. A thorough phylogenetic analysis of the sequence conservation, distribution and gene context of proteins containing the Csp fold may provide evolutionary insights into its function.

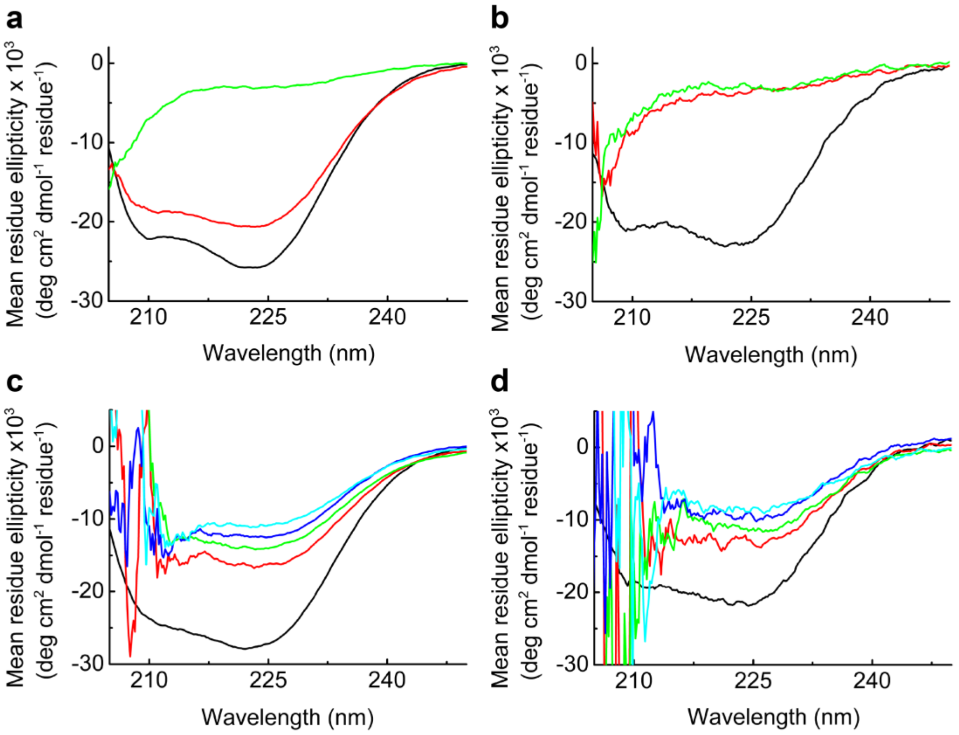

Supplementary Figure S2. Unfolding of *Mt*Csp3 monitored by far-UV CD spectroscopy. Far-UV CD spectra of apo-*Mt*Csp3 at (**a**) 27.5 µM (0.40 mg/ml) and (**b**) at 6.44 µM (0.094 mg/ml), 5 (**a**) and 15 (**b**) (red lines) and 60 (green lines) min after the addition of urea (6.7 M). Also shown are spectra of *Mt*Csp3 (29.1 µM, 0.42 mg/ml (**c**) and 7.28 µM, 0.11 mg/ml (**d**)) loaded with 17.4 equivalents of Cu(I), 15 min (red lines), 1 (green lines), 2 (blue lines) and 24 (cyan lines) h after the addition of guanidine hydrochloride (6.2 M). All data were acquired in 20 mM Hepes pH 7.5 containing 200 mM NaCl and in each case the spectrum drawn with a black line is that of the protein in the absence of denaturant.

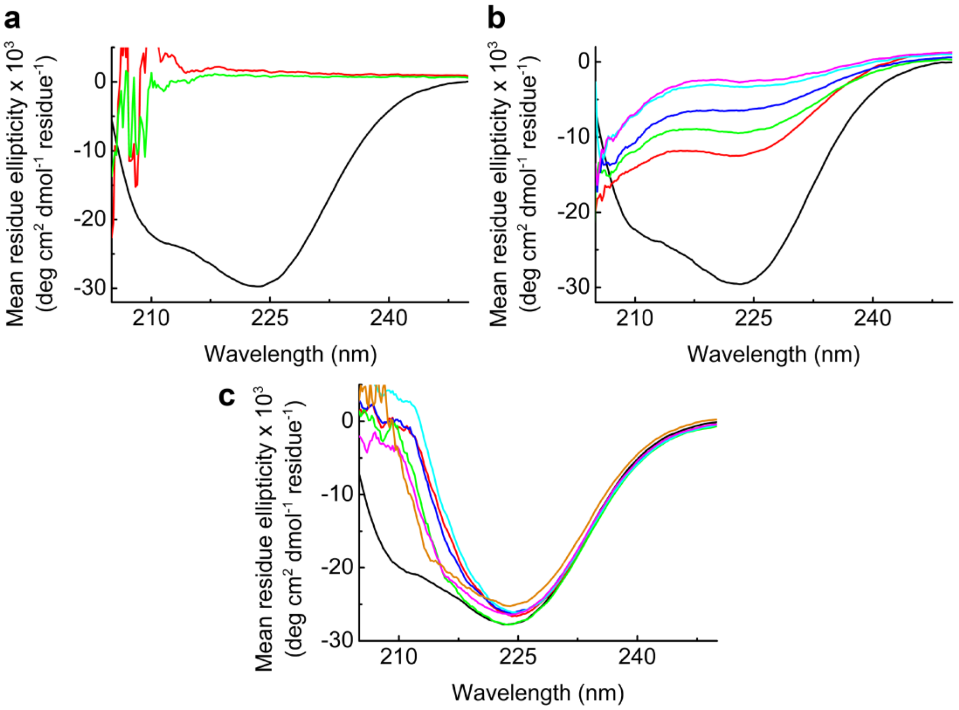

Supplementary Figure S3. Unfolding of *Bs*Csp3 monitored by far-UV CD spectroscopy. Far-UV CD spectra of (**a**) apo-*Bs*Csp3 (41.9 µM, 0.50 mg/ml), 5 (red line) and 40 (green line) min after the addition of guanidine hydrochloride (6.0 M). The spectra shown in (**b**) are for apo-*Bs*Csp3 (40.5 µM, 0.48 mg/ml), 20 min (red line), 1 (green line), 2 (blue line), 4 (cyan line) and 6 (magenta line) h after the addition of urea (6.7 M). The spectra in (**c**) are of *Bs*Csp3 (47.6 µM, 0.56 mg/ml) loaded with 19.2 equivalents of Cu(I), 1 (red line), 2 (green line), 4 (blue line), 6 (cyan line), 21 (magenta line) and 42 (orange line) h after the addition of guanidine hydrochloride (6.0 M). In all cases the protein was in 20 mM Hepes pH 7.5 plus 200 mM NaCl and spectra in this buffer with no denaturant are shown as black lines.

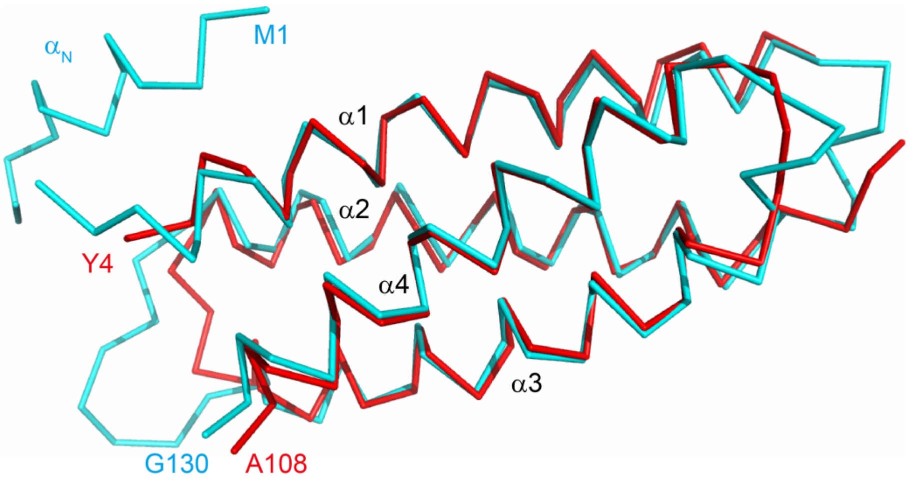

Supplementary Figure S4. Structure comparison of the Csp3s. Overlay of the C^{α} atoms (rmsd 1.09 Å for 103 aligned C^{α} atoms) for apo-*Mt*Csp3 (cyan) and apo-*Bs*Csp3 (red). α_N (and the loop to α1) is missing in *Bs*Csp3, and the loop between α2 and α3 is significantly shorter than in *Mt*Csp3 (3 rather than 7 residues). There are various degrees of completeness (disorder) in the 6 chains in the asymmetric unit of the apo-*Bs*Csp3 structure, with chain F missing 5 residues whilst 18 amino acids could not be modelled in chain D. The regions affected are the N-terminus (up to 3 residues), the loop between α1 and α2 (absent in chain D), and the loop between α3 and α4 (also absent in chain D). Therefore chain F (missing the first 3 residues plus Ser30 and Val31 from the loop between α1 and α2) has been used for the overlay shown above, and the tetramer in Fig. 1b consists of chains E and F and two symmetry related monomers. The side chains of Cys38 and Cys81 appear in alternate conformations in all chains except E (no evidence of disulfide) and D (complete disulfide formation), with ~30% disulfide estimated in the other four chains (in Fig. 1b the main conformation of Cys38 is shown for chain F). Cys81 is close to the C-terminus of α3 and at the end of the molecule where Cys residues are potentially more solvent exposed. Apo-*Bs*Csp3 was crystallised aerobically over approximately 3 weeks (the Bradford:DTNB ratio did not change over this time for a sample in solution treated with DTT and then incubated in air) in a relatively high concentration of ammonium sulfate at pH 4, which may cause partial unfolding at this end of the molecule. This is consistent with the observation of 100% Cys38-Cys81 disulfide in chain D in which the loop between Lys83 and His88, and Cys89 and Gln90, which are part of α4 in other chains, is disordered.

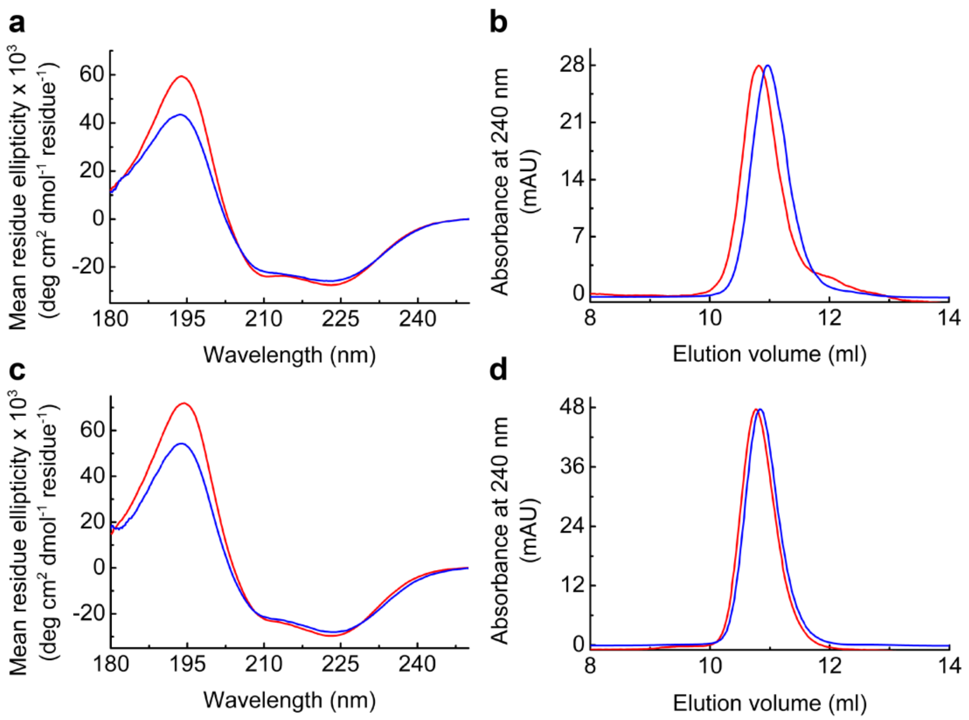

Supplementary Figure S5. Secondary and quaternary structures of apo- and Cu(I)-Csp3s. Far-UV CD spectra of (**a**) apo-*Mt*Csp3 (40.2 µM, 0.58 mg/ml, red line) and of *Mt*Csp3 (38.1 µM, 0.55 mg/ml) loaded with 18.1 equivalents of Cu(I) (blue line). (**b**) Analytical gel-filtration chromatograms of apo-*Mt*Csp3 (77.8 µM, red line) and *Mt*Csp3 (78.1 µM) to which 19.0 equivalents of Cu(I) were added (blue line). Apo-*Mt*Csp3 elutes mainly as a single peak with an apparent molecular weight of 45.2 ± 4.1 kDa (n = 31). A second peak with a greater elution volume (apparent molecular weight of 27.7 ± 2.0 kDa, n = 25) is more apparent at lower protein concentrations, but there is not a consistently reproducible dependence of the relative amounts of these on concentration. The addition of DTT did not have a significant effect on this behaviour. For Cu(I)-*Mt*Csp3 there is no sign of the smaller form of the protein even at low protein concentrations (~2 µM). (**c**) Far-UV CD spectra of apo-*Bs*Csp3 (41.4 µM, 0.49 mg/ml, red line) and *Bs*Csp3 loaded with 19.3 equivalents of Cu(I) (38.0 µM, 0.45 mg/ml, blue line). (**d**) Analytical gel-filtration chromatograms of apo-*Bs*Csp3 (112 µM, red line) and *Bs*Csp3 (92.0 µM) plus 19.1 equivalents of Cu(I) (blue line). Far-UV CD samples were in 100 mM phosphate pH 8.0, whilst gel-filtration chromatography was performed in 20 mM Hepes pH 7.5 plus either 200 (*Mt*Csp3) or 500 (*Bs*Csp3) mM NaCl. In (**b**) and (**d**) the absorbance was monitored at 240 nm with the values for Cu(I)-*Mt*Csp3 and Cu(I)-*Bs*Csp3 divided by 10 and 8.7 respectively.

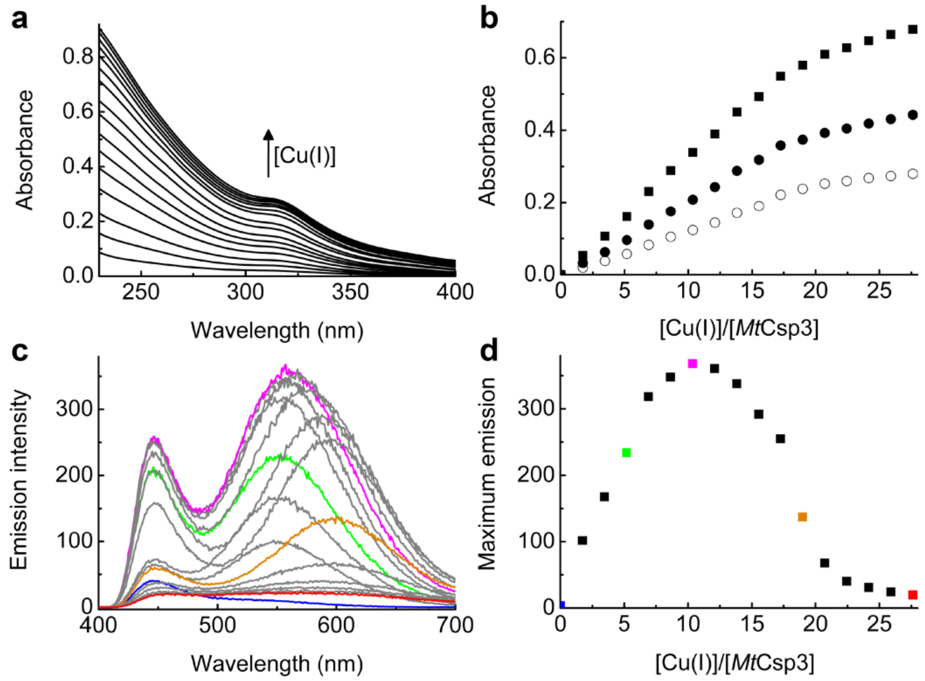

Supplementary Figure S6. *Mt*Csp3 Cu(I) binding monitored by UV-Vis and fluorescence spectroscopy. (**a**) UV-Vis difference spectra obtained upon Cu(I) titration into apo-*Mt*Csp3 (4.86 μM), and (**b**) plots of the absorbance at 250 (filled squares), 275 (filled circles) and 315 (open circles) nm against the [Cu(I)]/[*Mt*Csp3] ratio (for a monomer) for the spectra shown in (**a**). (**c**) Emission spectra after excitation at 280 nm upon the titration of Cu(I) into apo-*Mt*Csp3 (4.86 μM). (**d**) A plot of the maximum emission (between 550 and 600 nm) against the [Cu(I)]/[*Mt*Csp3] ratio (for a monomer) for spectra shown in (**c**). In (**c**) and (**d**) the data at the following number of Cu(I) equivalents are highlighted: 0 (blue); 5.18 (green); 10.4 (magenta); 19.0 (orange); 27.6 (red). Both experiments were performed in 20 mM Hepes plus 200 mM NaCl at pH 7.5.

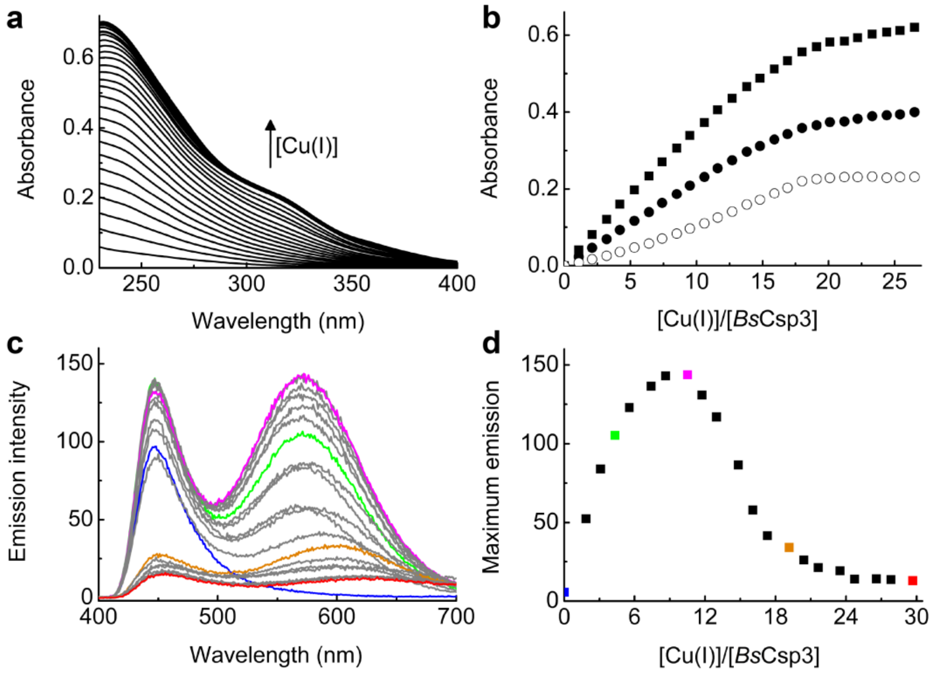

Supplementary Figure S7. *Bs*Csp3 Cu(I) binding monitored by UV-Vis and fluorescence spectroscopy. (**a**) UV-Vis difference spectra obtained upon Cu(I) titration into apo-*Bs*Csp3 (5.10 μM), and (**b**) plots of the absorbance at 250 (filled squares), 275 (filled circles) and 315 (open circles) nm against the [Cu(I)]/[*Bs*Csp3] ratio (for a monomer) for the spectra shown in (**a**). (**c**) Emission spectra after excitation at 310 nm upon the titration of Cu(I) into apo-*Bs*Csp3 (16.8 μM). (**d**) A plot of the maximum emission (between 550 and 600 nm) against the [Cu(I)]/[*Bs*Csp3] ratio (for a monomer) for spectra shown in (**c**). In (**c**) and (**d**) the data at the following number of Cu(I) equivalents are highlighted: 0 (blue); 4.33 (green); 10.5 (magenta); 19.2 (orange); 29.7 (red). Both experiments were performed in 20 mM Hepes plus 200 mM NaCl at pH 7.5.

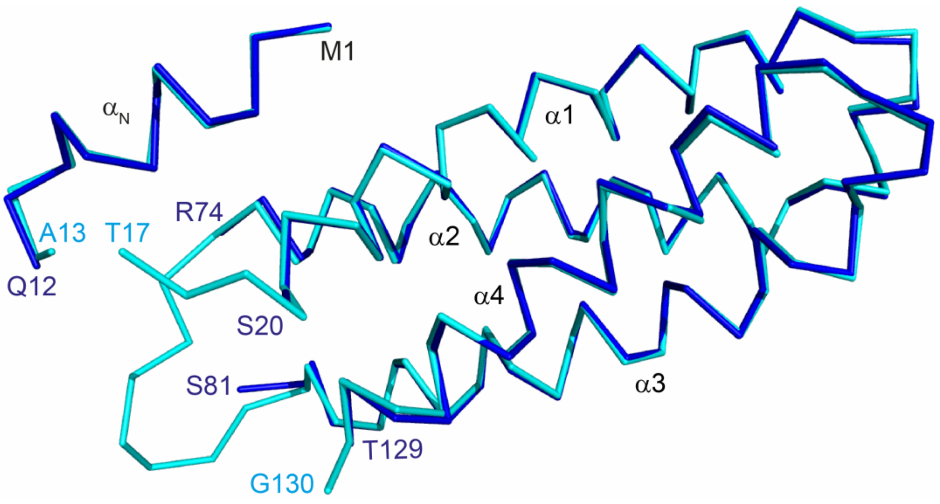

Supplementary Figure S8. Structure comparison of apo- and Cu(I)-*Mt*Csp3. An overlay of C^{α} atoms (rmsd 0.32 Å for 116 aligned C^{α} atoms) of the structures of apo-*Mt*Csp3 (cyan) and Cu(I)-*Mt*Csp3 (blue). There is evidence of disorder in the Cu(I)-*Mt*Csp3 structure, and the loops linking α_N and α1 (Ala13 to Arg19) and α2 and α3 (Ala75 to Glu80) are missing, as are the final 4 residues (final 3 in the apo-structure). The larger number of residues not modelled in the Cu(I)-*Mt*Csp3 crystal structure results in smaller calculated contact areas between monomers (ranging from ~830 to 1380 Å^2) than in the apo-*Mt*Csp3 tetramer (~1060 to 1410 Å^2). Crystals of Cu(I)-*Mt*Csp3 gave a well-defined peak due to the Cu 1s→4p transition, consistent with 2/3-coordinate Cu(I)[24].

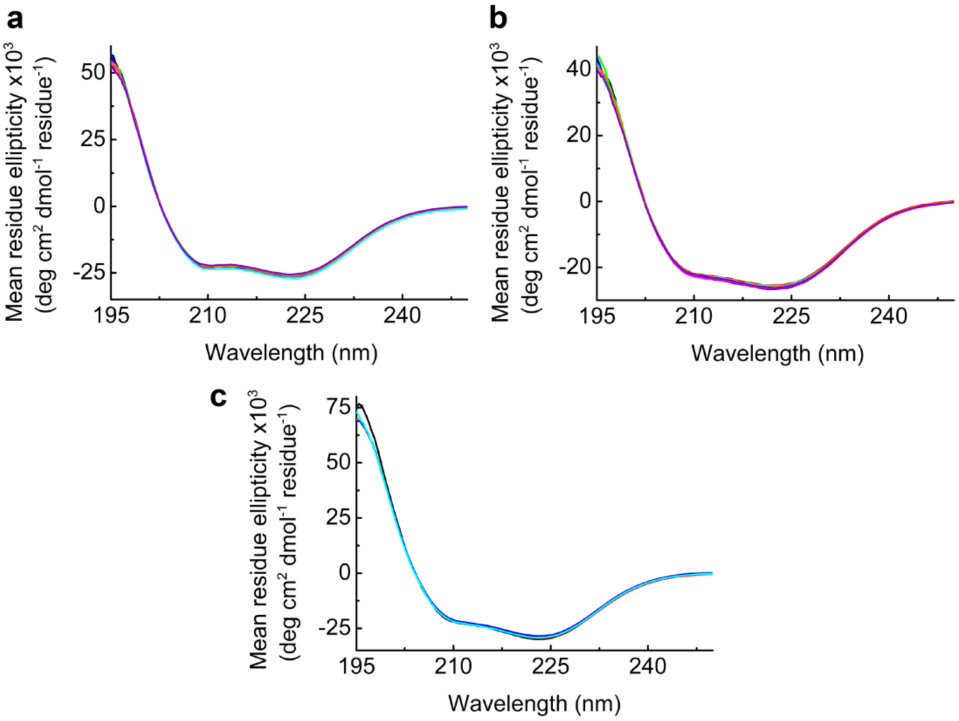

Supplementary Figure S9. The stability of Csp3s. Far-UV CD spectra of (**a**) apo-*Mt*Csp3 (41.4 µM, 0.60 mg/ml) and (**b**) *Mt*Csp3 (33.6 µM, 0.49 mg/ml) loaded with 16.9 equivalents of Cu(I), after incubation for 0 (black line), 23 (red line), 46 (green line), 70.5 (blue line), 94 (cyan line), 165 (magenta line), 234 (orange line) and 409 (violet line) h. (**c**) Spectra of apo-*Bs*Csp3 (43.9 µM, 0.52 mg/ml) incubated for 0 (black line), 24 (red line), 60 (green line), 132 (blue line), 228 (cyan line) h. Samples in 20 mM Hepes plus 200 mM NaCl at pH 7.5 were incubated at room temperature in the anaerobic chamber.

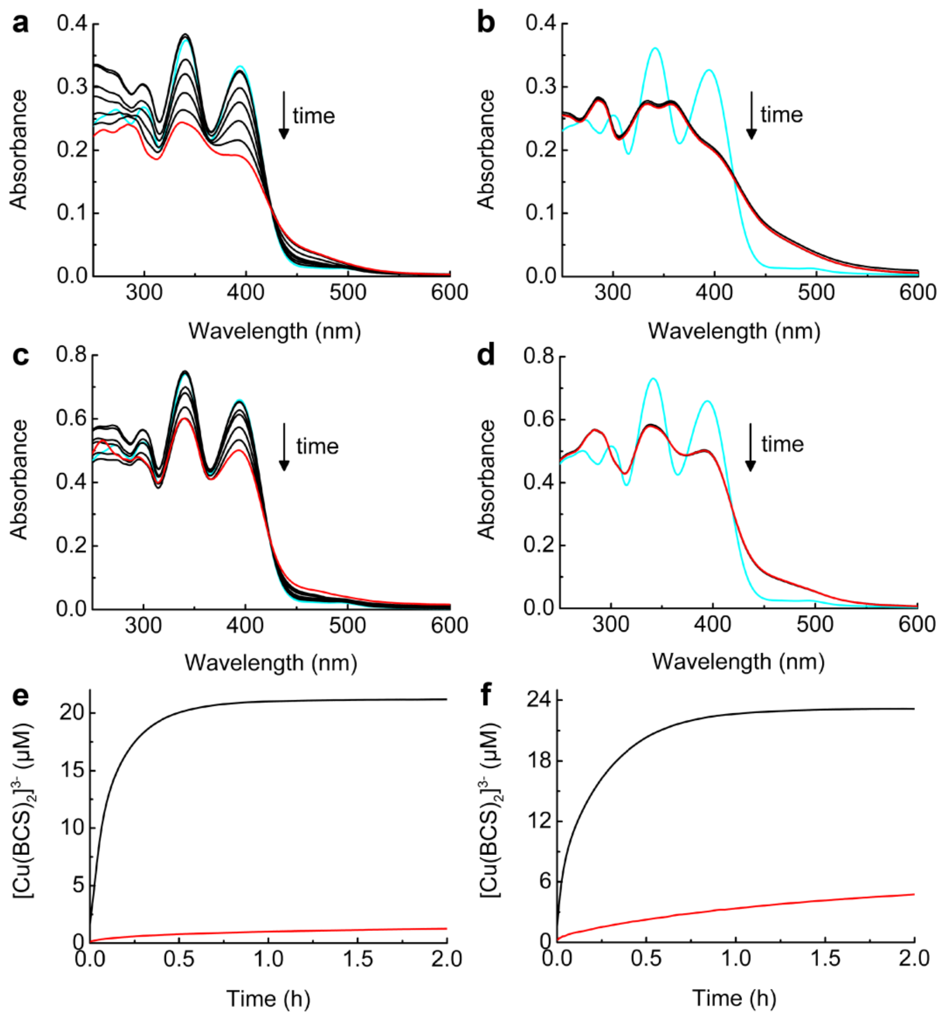

Supplementary Figure S10. Cu(I) removal from Csp3s. UV-Vis spectra recorded at different times (4, 20, 72, 144, 192, 312 and 360 h) after the addition of 0.96 (**a**) and 0.94 (**c**) μM *Mt*Csp3 loaded with 18.0 equivalents of Cu(I) to 17.1 (**a**) and 33.9 (**c**) μM apo-mbtin (cyan line). Also shown are spectra at different times (immediately post mixing and then after 1, 2 and 4 h) following the addition of 16.7 μM Cu(I) to 16.8 (**b**) and 33.8 (**d**) μM apo-mbtin (cyan line). The spectra after 360 h for the reaction with Cu(I)-*Mt*Csp3 and after 4 h with Cu(I) alone are shown in red. Also shown are plots of $[Cu(BCS)_2]^{3-}$ concentration against time (up to 2 h) for (**e**) 1.26 μM *Mt*Csp3 loaded with 16.6 equivalents of Cu(I) and (**f**) 1.24 μM *Bs*Csp3 loaded with 18.0 equivalents of Cu(I) mixed with 2.41 mM BCS in the absence (red line) and presence (black line) of 6.5 M guanidine hydrochloride. Experiments were performed in 20 mM Hepes plus 200 mM NaCl at pH 7.5.

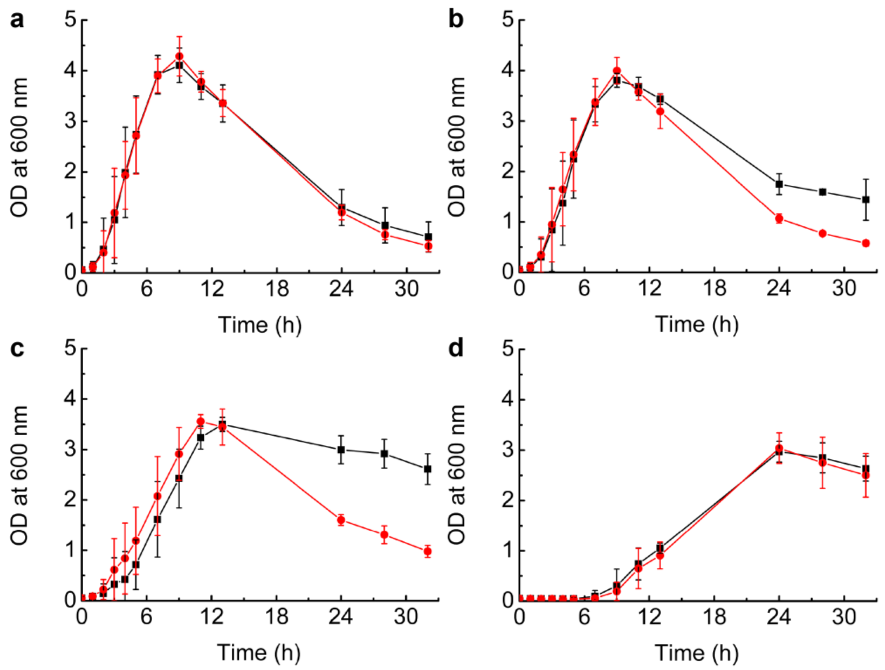

Supplementary Figure S11. Initial analysis of the Δ*csp3* strain of *B. subtilis*. Growth curves at 37 ºC of WT *B. subtilis* (black squares) and the Δ*csp3* strain (red circles) in LB media containing 1 (**a**), 1.5 (**b**), 2.0 (**c**) and 2.5 (**d**) mM copper nitrate. The averages and standard deviations from four independent growth experiments (three for the data at 28 and 32 h) are shown.

Supplementary Table S1. Data collection and refinement statistics.

	Apo-*Mt*Csp3<br>5ARM	Cu(I)-*Mt*Csp3<br>5ARN	Apo-*Bs*Csp3<br>5FIG
**Data collection***			
Beamline	I02	I02	I02
Wavelength (Å)	0.9796	1.377	0.9796
Resolution (Å)	34.27-1.19 (1.21-1.19)	41.91-2.30 (2.38-2.30)	45.41-1.70 (1.73-1.70)
Space group	C222	$I4_122$	$C222_1$
Unit-cell parameters			
a, *b*, *c* (Å)	45.2, 105.5,45.1	105.9, 105.9, 45.6	107.1, 126.5, 85.7
$\alpha = \beta = \gamma$ (°)		90	
Unit-cell volume ($Å^3$)	214778	511751	1161515
Solvent content (%)	37	51	40
No. of measured reflections	112434 (5369)	24815 (2442)	357889 (19100)
No. of independent reflections	34091 (1676)	5998 (570)	64008 (3365)
Completeness (%)	98.7 (99.1)	99.7 (99.9)	98.7 (98.0)
Redundancy	3.3 (3.2)	4.1 (4.3)	5.6 (5.7)
R_{merge} (%)	5.2 (43.9)	7.7 (65.3)	9.0 (71.0)
$\langle I\rangle/\langle\sigma(I)\rangle$	8.5 (2.2)	6.3 (1.9)	8.0 (1.5)
**Refinement statistics***			
R_{work} (%)	13.65	18.57	21.97
$R_{free}^{\#}$ (%)	17.10	24.27	24.26
No. of atoms			
Protein	965	838	4404
Cu atoms	0	19	0
Solvent	82	5	137
Average B-factors ($Å^2$)			
Protein	15.7	51.8	21.6
Solvent	27.0	51.9	23.5
Cu		46.8	
R.m.s. deviation from ideal values			
Bond angle (°)	1.9	1.5	1.8
Bond length (Å)	0.018	0.013	0.016
Ramachandran plot,[†] residues in most favoured regions (%)	100	100	100

* Values in parenthesis are for the highest resolution shell.
[#] 5% of the randomly selected reflections excluded from refinement.
[†] Calculated using MolProbity[21].
